# Supplementary material for: Integrating Proteomics and Lipidomics for Evaluating the Risk of Breast Cancer Progression: A Pilot Study
Source: Biomedicines. 2023 Jun 22;11(7):1786. doi: 10.3390/biomedicines11071786 (PMC10376786; doi:10.3390/biomedicines11071786)
Supplement: Supplementary file 1 [file biomedicines-11-01786-s001.zip › Supplementary S2.pdf]

Supplementary 2

TABLE OF CONTENT

Table S1. Transition list and parameters used for LC-MRM MS..2

Figure S1. The exemplary MRM data (from Skyline) and calibration curves of eleven potential marker serum proteins for BC metastatic progressio..10

Figure S2. LC-MS chromatogram for lipid extract of the sample with marked regions for each lipid class detected. 16

Figure S3. Corrplot of proteins and lipids correlated significantly with number of metastasis...17

Figure S4. Boxplot of protein and lipids concetrations, which are significantly different in serum in various histological types...18

Figure S5. Corrplot of proteins and lipids with significant correlation with BC grade. .19

Figure S6. Plot of impact of potential BC metastasis markers on metabolic pathways...20

Table S2. Metabolic pathways, disturbed by regional metastasis......................................21

Table S1. Transition list and parameters used for LC-MRM MS. Liquid chromatography - multiple reaction monitoring mass spectrometry (LC-MRM MS) was performed using QTRAP SCIEX6500+ mass spectrometer (SCIEX, Canada). The BAK 125 kit (MRM Proteomics Inc, Montreal, Canada) was used to quantify 125 proteins by measuring the concentration of one surrogate proteotypic peptide for each protein. All 125 peptides are measured within a single LC-MRM MS run. The observed ratio of the peak areas for the light peptide vs. the fixed-concentration SIS peptide were used to calculate the concentration of the endogenous peptide in serum.

Q1 mass	Q3 mass	RT	Peptide ID	DP	CE
788.898	681.832	23.54	78 kDa glucose-regulated protein.ITPSYVAFTPEGER.P11021.y12++.heavy	20	32
783.89	676.83	23.54	78 kDa glucose-regulated protein.ITPSYVAFTPEGER.P11021.y12++.light	20	32
560.3	893.424	15.3	Adipocyte plasma membrane-associated protein.LLEYDTVTR.Q9HDC9.y7.heavy	20	23
555.295	883.42	15.3	Adipocyte plasma membrane-associated protein.LLEYDTVTR.Q9HDC9.y7.light	20	23
591.273	756.329	8.1	Adiponectin.IFYNQQNHYDGSTGK.+3y13+2.light	60	26
593.939	760.34	8.28	Adiponectin.IFYNQQNHYDGSTGK.Q15848.y13++.heavy	60	23
567.764	833.428	19.83	Afamin.DADPDTFFAK.P43652.y7.heavy	20	27
563.764	825.428	19.83	Afamin.DADPDTFFAK.P43652.y7.light	20	27
572.962	708.877	20.7	Alpha-1-acid glycoprotein 1.NWGLSVYADKPETTK.P02763.y13++.heavy	20	23
570.295	704.877	20.7	Alpha-1-acid glycoprotein 1.NWGLSVYADKPETTK.P02763.y13++.light	20	23
535.305	252.18	24.03	Alpha-1-antichymotrypsin.EIGELYLPK.P01011.y2.heavy	20	24
531.305	244.18	24.03	Alpha-1-antichymotrypsin.EIGELYLPK.P01011.y2.light	20	24
559.813	805.419	20.1	Alpha-1-antitrypsin .LSITGTYDLK.PEP2013073114.2.y7.heavy	20	35
555.813	797.419	20.1	Alpha-1-antitrypsin .LSITGTYDLK.PEP2013073114.2.y7.light	20	35
623.334	243.134	34.3	Alpha-1B-glycoprotein.LETPDFQLFK.P04217.b2.heavy	20	31
619.334	243.134	34.3	Alpha-1B-glycoprotein.LETPDFQLFK.P04217.b2.light	20	31
660.854	542.257	7	Alpha-2-antiplasmin.LGNQEPGGQTALK.+2b5.heavy	60	31.2
656.846	542.257	7	Alpha-2-antiplasmin.LGNQEPGGQTALK.+2b5.light	60	31.2
411.237	587.364	13.32	Alpha-2-HS-glycoprotein.FSVVYAK.P02765.y5.heavy	20	19
407.229	579.349	13.32	Alpha-2-HS-glycoprotein.FSVVYAK.P02765.y5.light	20	19
633.33	861.445	16	Alpha-2-macroglobulin.AIGYLNTGYQR.P01023.y7.heavy	20	31
628.33	851.445	16	Alpha-2-macroglobulin.AIGYLNTGYQR.P01023.y7.light	20	31
439.879	487.745	17.72	Antithrombin-III.DDLYVSDAFHK.P01008.y8++.heavy	20	19
437.21	483.74	17.72	Antithrombin-III.DDLYVSDAFHK.P01008.y8++.light	20	19
408.551	576.803	5.43	Apolipoprotein A-I.ATEHLSTLSEK.P02647.y10++.heavy	20	21
405.88	572.8	5.43	Apolipoprotein A-I.ATEHLSTLSEK.P02647.y10++.light	20	21
475.294	478.348	18	Apolipoprotein A-II .EQLTPLIK.PEP20130306.2.y4.heavy	20	23
471.295	470.348	18	Apolipoprotein A-II .EQLTPLIK.PEP20130306.2.y4.light	20	23
708.367	300.155	16.11	Apolipoprotein A-IV.LGEVNTYAGDLQK.P06727.b3.heavy	40	35
704.36	300.155	16.11	Apolipoprotein A-IV.LGEVNTYAGDLQK.P06727.b3.light	40	35
528.297	454.763	25.2	Apolipoprotein B-100.FPEVDVLTK.P04114.y8++.heavy	20	28
524.29	450.763	25.2	Apolipoprotein B-100.FPEVDVLTK.P04114.y8++.light	20	28
605.288	894.445	23.86	Apolipoprotein C-I.EWFSETFQK.P02654.y7.heavy	20	29
601.28	886.43	23.86	Apolipoprotein C-I.EWFSETFQK.P02654.y7.light	20	29

522.279	265.118	13.95	Apolipoprotein C-II.TYLPAVDEK.P02655.b2.heavy	40	23
518.27	265.118	13.95	Apolipoprotein C-II.TYLPAVDEK.P02655.b2.light	40	23
602.809	244.108	27.25	Apolipoprotein C-III.GWVTDGFSSLK.P02656.b2.heavy	40	27
598.809	244.108	27.25	Apolipoprotein C-III.GWVTDGFSSLK.P02656.b2.light	40	27
541.808	497.308	18.97	Apolipoprotein C-IV.ELLETVVNR.P55056.y4.heavy	40	22
536.808	487.308	18.97	Apolipoprotein C-IV.ELLETVVNR.P55056.y4.light	40	27
441.258	669.356	6.6	Apolipoprotein D .VLNQELR.PEP2013021305.2.y5.heavy	40	40
436.258	659.356	6.6	Apolipoprotein D .VLNQELR.PEP2013021305.2.y5.light	40	40
489.74	404.732	11.85	Apolipoprotein E.LGPLVEQGR.P02649.y7++.heavy	20	23
484.78	399.732	11.85	Apolipoprotein E.LGPLVEQGR.P02649.y7++.light	20	23
477.256	783.397	4.4	Apolipoprotein L1.VAQELEEK.O14791.y6.heavy	20	22
473.249	775.397	4.4	Apolipoprotein L1.VAQELEEK.O14791.y6.light	20	23
414.255	609.396	20.89	Apolipoprotein M.AFLLTPR.O95445.y5.heavy	20	19
409.251	599.396	20.89	Apolipoprotein M.AFLLTPR.O95445.y5.light	20	19
658.361	720.449	21.5	Apolipoprotein(a) .TPAYYPNAGLIK.PEP2014012820.2.y7.heavy	20	25
654.35	712.43	21.5	Apolipoprotein(a) .TPAYYPNAGLIK.PEP2014012820.2.y7.light	20	25
448.764	710.418	6.44	Attractin.SVNNVVVR.O75882.y6.heavy	20	23
443.759	700.409	6.44	Attractin.SVNNVVVR.O75882.y6.light	20	23
516.772	761.382	5.61	Beta-2-glycoprotein 1.ATVVYQGER.P02749.y6.heavy	40	27
511.772	751.382	5.61	Beta-2-glycoprotein 1.ATVVYQGER.P02749.y6.light	40	27
624.36	578.38	23.63	Beta-Ala-His dipeptidase.ALEQDLPVNIK.Q96KN2.y5.heavy	40	27
620.36	570.38	23.63	Beta-Ala-His dipeptidase.ALEQDLPVNIK.Q96KN2.y5.light	40	27
363.229	451.266	13.3	Biotinidase.SHLIIAQVAK.P43251.b4.heavy	20	18
360.557	451.266	13.3	Biotinidase.SHLIIAQVAK.P43251.b4.light	20	18
643.833	228.134	6.97	Cadherin-13.INENTGSVSVTR.P55290.b2.heavy	20	31
638.83	228.134	6.97	Cadherin-13.INENTGSVSVTR.P55290.b2.light	20	31
489.81	766.46	22.94	Carbonic anhydrase 1.VLDALQAIK.P00915.y7.heavy	40	19
485.81	758.46	22.94	Carbonic anhydrase 1.VLDALQAIK.P00915.y7.light	40	19
302.185	267.673	14.6	Carboxypeptidase B2.IAWHVIR.+3y4+2.heavy	20	12.3
298.848	262.669	14.6	Carboxypeptidase B2.IAWHVIR.+3y4+2.light	20	12.3
448.745	712.362	5.58	Cathelicidin antimicrobial peptide.AIDGINQR.P49913.y6.heavy	40	22
443.745	702.362	5.58	Cathelicidin antimicrobial peptide.AIDGINQR.P49913.y6.light	40	22
407.871	467.26	6.6	Cation-independent mannose-6-phosphate receptor.GHQAFDVGQPR.+3y4.heavy	20	17.4
404.535	457.252	6.6	Cation-independent mannose-6-phosphate receptor.GHQAFDVGQPR.+3y4.light	20	17.4
381.237	549.313	4.87	CD5 antigen-like.LVGGLHR.O43866.y5.heavy	20	23
376.237	539.313	4.87	CD5 antigen-like.LVGGLHR.O43866.y5.light	20	23
396.88	456.743	4.85	Ceruloplasmin.IYHSHIDAPK.P00450.y8++.heavy	40	19
394.21	452.74	4.85	Ceruloplasmin.IYHSHIDAPK.P00450.y8++.light	40	19
604.314	931.472	13.01	Cholinesterase.YLTLNTESTR.P06276.y8.heavy	20	30
599.314	921.472	13.01	Cholinesterase.YLTLNTESTR.P06276.y8.light	20	30
649.827	385.207	15.11	Clusterin.ELDESLQVAER.P10909.y3.heavy	40	27

644.823	375.207	15.11	Clusterin.ELDESLQVAER.P10909.y3.light	40	27
536.824	702.417	29.4	Coagulation factor IX.SALVLQYLR.P00740.y5.heavy	40	27
531.82	692.417	29.4	Coagulation factor IX.SALVLQYLR.P00740.y5.light	40	27
607.272	997.462	8	Coagulation factor V .SEAYNTFSER.PEP2013021902.2.y8.heavy	20	27
602.272	987.462	8	Coagulation factor V .SEAYNTFSER.PEP2013021902.2.y8.light	20	27
378.542	442.237	5.3	Coagulation factor VIII.LHPTHYSIR.P00451.y7++.heavy	40	18
375.21	437.23	5.3	Coagulation factor VIII.LHPTHYSIR.P00451.y7++.light	40	18
452.25	533.271	13.8	Coagulation factor X .TGIVSGFGR.PEP2013020903.2.y5.heavy	60	30
447.25	523.271	13.8	Coagulation factor X .TGIVSGFGR.PEP2013020903.2.y5.light	60	30
469.253	340.702	6.51	Coagulation factor XII.EQPPSLTR.P00748.y6++.heavy	20	22
464.248	335.697	6.51	Coagulation factor XII.EQPPSLTR.P00748.y6++.light	20	22
388.221	591.313	8.84	Complement C1q subcomponent subunit B.IAFSATR.P02746.y5.heavy	20	19
383.221	581.313	8.84	Complement C1q subcomponent subunit B.IAFSATR.P02746.y5.light	20	19
547.797	819.46	20.4	Complement C1q subcomponent subunit C.FQSVFTVTR.+2y7.heavy	40	25.6
542.793	809.452	20.4	Complement C1q subcomponent subunit C.FQSVFTVTR.+2y7.light	40	25.6
263.841	310.205	11.8	Complement C1r subcomponent.GLTLHLK.P00736.y5++.heavy	20	10.5
261.175	306.205	11.8	Complement C1r subcomponent.GLTLHLK.P00736.y5++.light	20	10.5
332.183	448.237	5.1	Complement C1r subcomponent-like protein.VVVHPDYR.+3y7+2.heavy	20	13.8
328.847	443.233	5.1	Complement C1r subcomponent-like protein.VVVHPDYR.+3y7+2.light	20	13.8
644.333	216.098	23.1	Complement C1s subcomponent.TNFDNDIALVR.P09871.b2.heavy	60	32
639.333	216.098	23.1	Complement C1s subcomponent.TNFDNDIALVR.P09871.b2.light	60	32
360.87	469.26	11.5	Complement C2.HAFILQDTK.P06681.b4.heavy	20	23
358.2	469.26	11.5	Complement C2.HAFILQDTK.P06681.b4.light	20	23
505.785	739.408	10.92	Complement C3.TGLQEVEVK.P01024.y6.heavy	20	27
501.785	731.408	10.92	Complement C3.TGLQEVEVK.P01024.y6.light	20	27
459.762	672.381	23.2	Complement C5.VFQFLEK.+2y5.heavy	20	21.3
455.755	664.366	23.2	Complement C5.VFQFLEK.+2y5.light	20	21.3
756.371	227.175	23.3	Complement component C7 .LIDQYGTHYLQSGSLGGEYR.PEP20120919025.3.b2.heavy	60	26.7
753.038	227.175	23.3	Complement component C7 .LIDQYGTHYLQSGSLGGEYR.PEP20120919025.3.b2.light	60	26.7
625.884	525.826	32.6	Complement component C9.LSPIYNLVPVK.P02748.y9++.heavy	20	27
621.884	521.826	32.6	Complement component C9.LSPIYNLVPVK.P02748.y9++.light	20	27
582.324	679.387	18.85	Complement factor B.EELLPAQDIK.P00751.y6.heavy	20	23
578.324	671.387	18.85	Complement factor B.EELLPAQDIK.P00751.y6.light	20	23

401.522	514.748	2.6	Complement factor H.SSQESYAHGTK.+3y9+2.heavy	20	17.1
398.851	510.741	2.6	Complement factor H.SSQESYAHGTK.+3y9+2.light	20	17.1
600.829	954.514	30.2	Complement factor I.VFSLQWGEVK.P05156.y8.heavy	20	23
596.829	946.514	30.2	Complement factor I.VFSLQWGEVK.P05156.y8.light	20	23
886.969	252.18	34	Corticosteroid-binding globulin.WSAGLTSSQVDLYIPK.P08185.y2.heavy	20	39
882.962	244.166	34	Corticosteroid-binding globulin.WSAGLTSSQVDLYIPK.P08185.y2.light	20	39
617.813	300.155	21.5	Cystatin-C.ALDFAVGEYNK.+2b3.heavy	20	29.1
613.806	300.155	21.5	Cystatin-C.ALDFAVGEYNK.+2b3.light	20	29.1
521.321	827.502	33.2	Endothelial protein C receptor.TLAFPLTIR.Q9UNN8.y7.heavy	20	23
516.321	817.502	33.2	Endothelial protein C receptor.TLAFPLTIR.Q9UNN8.y7.light	20	23
460.807	708.453	31.4	Fetuin-B.LVVLPFPK.Q9UGM5.y6.heavy	20	19
456.807	700.453	31.4	Fetuin-B.LVVLPFPK.Q9UGM5.y6.light	20	19
549.93	516.281	8.7	Fibrinogen alpha chain .ESSSHHPGIAEFPSR.PEP9999032685.3.y4.heavy	20	31
546.597	506.281	8.7	Fibrinogen alpha chain .ESSSHHPGIAEFPSR.PEP9999032685.3.y4.light	20	31
713.036	764.376	25.4	Fibrinogen beta chain.HQLYIDETVNSNIPTNLR.P02675.b13++.heavy	20	19
709.703	764.376	25.4	Fibrinogen beta chain.HQLYIDETVNSNIPTNLR.P02675.b13++.light	20	19
501.256	605.327	15.5	Fibrinogen gamma chain.YEASILTHDSSIR.P02679.y11++.heavy	20	23
497.92	600.32	15.5	Fibrinogen gamma chain.YEASILTHDSSIR.P02679.y11++.light	20	23
625.303	744.392	11.5	Fibronectin.HTSVQTTSSGSGPFTDVR.P02751.y6.heavy	40	23
621.969	734.392	11.5	Fibronectin.HTSVQTTSSGSGPFTDVR.P02751.y6.light	40	23
594.782	704.36	19.82	Fibulin-1.TGYYFDGISR.P23142.y6.heavy	20	27
589.782	694.36	19.82	Fibulin-1.TGYYFDGISR.P23142.y6.light	20	27
682.4	878.544	35.2	Galectin-3-binding protein.SDLAVPSELALLK.Q08380.y8.heavy	20	28
678.4	870.544	35.2	Galectin-3-binding protein.SDLAVPSELALLK.Q08380.y8.light	20	28
664.359	200.103	22.2	Gelsolin.AGALNSNDAFVLK.P06396.b3.heavy	40	35
660.359	200.103	22.2	Gelsolin.AGALNSNDAFVLK.P06396.b3.light	40	35
781.912	653.361	22.66	Glutathione peroxidase 3.QEPGENSEILPTLK.P22352.y12++.heavy	20	35
777.912	649.361	22.66	Glutathione peroxidase 3.QEPGENSEILPTLK.P22352.y12++.light	20	35
649.877	500.302	31.44	Haptoglobin.DIAPTLTLYVGK.P00738.y9++.heavy	20	24
645.877	496.302	31.44	Haptoglobin.DIAPTLTLYVGK.P00738.y9++.light	20	24
513.92	498.29	10.1	Hemoglobin subunit alpha.VGAHAGEYGAEALER.P69905.y4.heavy	20	24
510.59	488.29	10.1	Hemoglobin subunit alpha.VGAHAGEYGAEALER.P69905.y4.light	20	24
615.811	485.255	26.3	Hemopexin.NFPSPVDAAFR.P02790.y9++.heavy	40	23
610.811	480.255	26.3	Hemopexin.NFPSPVDAAFR.P02790.y9++.light	40	23

519.795	824.451	13.2	Heparin cofactor 2.TLEAQLTPR.P05546.y7.heavy	20	27
514.795	814.451	13.2	Heparin cofactor 2.TLEAQLTPR.P05546.y7.light	20	27
599.827	901.477	27.4	Hepatocyte growth factor-like protein.SPLNDFQVLR.P26927.y7.heavy	20	32
594.827	891.477	27.4	Hepatocyte growth factor-like protein.SPLNDFQVLR.P26927.y7.light	20	32
497.787	796.429	12.64	Hyaluronan-binding protein 2.VVLGDQDLK.Q14520.y7.heavy	20	22
493.787	788.429	12.64	Hyaluronan-binding protein 2.VVLGDQDLK.Q14520.y7.light	20	22
393.231	291.186	19.4	Ig mu chain C region.GFPSVLR.P01871.y5++.heavy	20	17
388.227	286.181	19.4	Ig mu chain C region.GFPSVLR.P01871.y5++.light	20	17
559.935	737.354	15.94	Insulin-like growth factor I.GFYFNKPTGYGSSSR.P05019.y13++.heavy	20	23
556.602	732.354	15.94	Insulin-like growth factor I.GFYFNKPTGYGSSSR.P05019.y13++.light	20	23
419.25	566.34	5.14	Inter-alpha-trypsin inhibitor heavy chain H2.SLAPTAAAK.P19823.y6.heavy	20	19
415.25	558.34	5.14	Inter-alpha-trypsin inhibitor heavy chain H2.SLAPTAAAK.P19823.y6.light	20	19
544.844	862.513	29.5	Intercellular adhesion molecule 1.LLGIETPLPK.P05362.y8.heavy	40	26
540.844	854.513	29.5	Intercellular adhesion molecule 1.LLGIETPLPK.P05362.y8.light	40	26
397.219	568.318	6.83	Interleukin-10.AHVNSLGENLK.P22301.y5.heavy	20	17
394.55	560.3	6.83	Interleukin-10.AHVNSLGENLK.P22301.y5.light	20	17
431.919	597.84	20.1	Kallistatin.VGSALFLSHNLK.P29622.y11++.heavy	20	21
429.252	593.84	20.1	Kallistatin.VGSALFLSHNLK.P29622.y11++.light	20	21
636.807	201.123	7.4	Keratin type I cytoskeletal 10.SLLEGEGSSGGGGR.P13645.b2.heavy	20	35
631.802	201.124	7.4	Keratin type I cytoskeletal 10.SLLEGEGSSGGGGR.P13645.b2.light	20	35
602.316	293.113	16.69	Keratin,type II cytoskeletal.YEELQVTVGR.PEP20121106047.2.b2.heavy	20	30
597.312	293.114	16.69	Keratin,type II cytoskeletal.YEELQVTVGR.PEP20121106047.2.b2.light	20	30
630.306	1059.487	20	Kininogen-1 .TVGSDTFYSFK.PEP2015082124.2.y9.heavy	20	23
626.306	1051.487	20	Kininogen-1 .TVGSDTFYSFK.PEP2015082124.2.y9.light	20	23
595.345	735.402	30.36	Leucine-rich alpha-2-glycoprotein.DLLLPQPDLR.P02750.y6.heavy	20	24
590.345	725.402	30.36	Leucine-rich alpha-2-glycoprotein.DLLLPQPDLR.P02750.y6.light	20	24
629.336	930.447	33.8	Lipopolysaccharide-binding protein.ITLPDFTGDLR.P18428.y8.heavy	20	27
624.336	920.447	33.8	Lipopolysaccharide-binding protein.ITLPDFTGDLR.P18428.y8.light	20	27
501.766	802.444	13.3	L-selectin.AEIEYLEK.P14151.y6.heavy	40	19
497.76	794.43	13.3	L-selectin.AEIEYLEK.P14151.y6.light	40	19
399.718	541.312	19.87	Lysozyme C.AWVAWR.P61626.y4.heavy	20	19
394.714	531.303	19.87	Lysozyme C.AWVAWR.P61626.y4.light	20	19

820.924	258.145	26.58	Mannan-binding lectin serine protease 1.TGVITSPDFPNPYPK.P48740.b3.heavy	60	40
816.917	258.145	26.58	Mannan-binding lectin serine protease 1.TGVITSPDFPNPYPK.P48740.b3.light	60	40
499.261	585.338	24.8	Mannan-binding lectin serine protease 2.WPEPVFGR.O00187.y5.heavy	20	27
494.256	575.329	24.8	Mannan-binding lectin serine protease 2.WPEPVFGR.O00187.y5.light	20	27
401.233	283.186	10.3	Metalloproteinase inhibitor 2.EYLIAGK.+2y3.heavy	20	18.5
397.226	275.171	10.3	Metalloproteinase inhibitor 2.EYLIAGK.+2y3.light	20	18.5
434.263	544.814	17.5	Myeloblastin.LVNVVLGAHNVR.P24158.y10++.heavy	20	15
430.93	539.814	17.5	Myeloblastin.LVNVVLGAHNVR.P24158.y10++.light	20	15
398.568	476.777	8.4	Paraoxonase(PON3).IQNVLSEKPR.S-100205-00004.Q15166.y8++.heavy	20	19
395.235	471.777	8.4	Paraoxonase(PON3).IQNVLSEKPR.S-100205-00004.Q15166.y8++.light	20	19
435.76	700.41	26.13	Peroxiredoxin-2.GLFIIDGK.P32119.y6.heavy	20	14
431.76	692.41	26.13	Peroxiredoxin-2.GLFIIDGK.P32119.y6.light	20	14
495.287	498.327	20.74	Phosphatidylinositol-glycan-specific phospholipase D.FGSSLITVR.P80108.y4.heavy	40	23
490.282	488.319	20.74	Phosphatidylinositol-glycan-specific phospholipase D.FGSSLITVR.P80108.y4.light	40	23
669.333	519.758	8.87	Phospholipid transfer protein.AVEPQLQEEER.P55058.y8++.heavy	40	32
664.333	514.758	8.87	Phospholipid transfer protein.AVEPQLQEEER.P55058.y8++.light	40	35
696.351	1150.551	27.5	Pigment epithelium-derived factor.LQSLFDSPDFSK.P36955.y10.heavy	40	32
692.351	1142.551	27.5	Pigment epithelium-derived factor.LQSLFDSPDFSK.P36955.y10.light	40	32
598.358	920.581	32	Plasma protease C1 inhibitor.FQPTLLTLPR.P05155.y8.heavy	40	22
593.358	910.581	32	Plasma protease C1 inhibitor.FQPTLLTLPR.P05155.y8.light	40	22
586.296	501.243	35.1	Plasma serine protease inhibi.AVVEVDESGTR.PEP9999032429.2.y9++.heavy	20	23
581.296	496.243	35.1	Plasma serine protease inhibi.AVVEVDESGTR.PEP9999032429.2.y9++.light	20	23
443.258	261.16	17.5	Plasminogen .LFLEPTR.PEP2014090309.2.b2.heavy	20	27
438.254	261.16	17.5	Plasminogen .LFLEPTR.PEP2014090309.2.b2.light	20	27
557.311	512.292	7.2	Plasminogen activator inhibit.VFQQVAQASK.PEP2014012801.2.y5.heavy	20	23
553.3	504.28	7.2	Plasminogen activator inhibit.VFQQVAQASK.PEP2014012801.2.y5.light	20	23
556.326	669.402	17.8	Pregnancy zone protein.ISEITNIVSK.+2y6.heavy	20	26.1
552.319	661.388	17.8	Pregnancy zone protein.ISEITNIVSK.+2y6.light	20	26.1
485.271	815.416	11.47	Protein AMBP.HHGPTITAK.+2b8.heavy	40	22.6
481.264	815.416	11.47	Protein AMBP.HHGPTITAK.+2b8.light	40	22.6
443.25	657.38	22.81	Protein S100-A9.DLQNFLK.P06702.y5.heavy	20	19
439.25	649.38	22.81	Protein S100-A9.DLQNFLK.P06702.y5.light	20	14

640.83	702.418	32	Protein Z-dependent protease inhibitor.ETSNFGFSLLR.Q9UK55.y6.heavy	20	27
635.83	692.418	32	Protein Z-dependent protease inhibitor.ETSNFGFSLLR.Q9UK55.y6.light	20	27
602.808	720.355	23.64	Prothrombin.ELLESYIDGR.P00734.y6.heavy	60	26
597.808	710.355	23.64	Prothrombin.ELLESYIDGR.P00734.y6.light	60	26
603.824	857.497	33.3	Retinol-binding protein 4.YWGVASFLQK.P02753.y8.heavy	20	28
599.824	849.497	33.3	Retinol-binding protein 4.YWGVASFLQK.P02753.y8.light	20	28
493.756	743.418	13.53	Serotransferrin.DGAGDVAFVK.P02787.y7.heavy	60	23
489.756	735.418	13.53	Serotransferrin.DGAGDVAFVK.P02787.y7.light	60	23
579.319	226.164	18.74	Serum albumin.LVNEVTEFAK.P02768.y2.heavy	40	47
575.319	218.164	18.74	Serum albumin.LVNEVTEFAK.P02768.y2.light	40	47
947.466	878.497	32.5	Serum paraoxonase_arylesterase 1.IFFYDSENPPASEVLR.+2y8.heavy	20	40
942.462	868.489	32.5	Serum paraoxonase_arylesterase 1.IFFYDSENPPASEVLR.+2y8.light	20	40
345.886	405.242	20.13	Serum paraoxonase_lactonase 3.ILIGTVFHK.+3y7+2.heavy	40	14.5
343.215	401.235	20.13	Serum paraoxonase_lactonase 3.ILIGTVFHK.+3y7+2.light	40	14.5
477.261	594.325	18.8	SPARC.LEAGDHPVELLAR.P09486.y11++.heavy	20	21
473.928	589.325	18.8	SPARC.LEAGDHPVELLAR.P09486.y11++.light	20	21
531.261	813.398	11.58	Tenascin.FTTDLDSPR.P24821.y7.heavy	20	22
526.261	803.398	11.58	Tenascin.FTTDLDSPR.P24821.y7.light	20	22
728.408	227.176	27.76	Tenascin-X.ILISGLEPSTPYR.P22105.b2.heavy	20	36
723.408	227.176	27.76	Tenascin-X.ILISGLEPSTPYR.P22105.b2.light	20	36
441.768	498.292	19.07	Thrombospondin-1.GTLLALER.P07996.y4.heavy	20	25
436.768	488.292	19.07	Thrombospondin-1.GTLLALER.P07996.y4.light	20	25
418.899	526.296	22.6	Thrombospondin-4.KPQDFLEELK.P35443.y4.heavy	20	20
416.233	518.296	22.6	Thrombospondin-4.KPQDFLEELK.P35443.y4.light	20	20
292.179	352.713	6.6	Thyroxine-binding globulin.AVLHIGEK.+3y6+2.heavy	20	11.9
289.508	348.706	6.6	Thyroxine-binding globulin.AVLHIGEK.+3y6+2.light	20	11.9
549.79	788.439	19.27	Tissue factor pathway inhibitor (isoform 1).FYYNSVIGK.P10646-1.y7.heavy	40	24
545.79	780.439	19.27	Tissue factor pathway inhibitor (isoform 1).FYYNSVIGK.P10646-1.y7.light	40	24
511.766	824.388	3.5	Tissue-type plasminogen activator.VVPGEEEQK.P00750.y7.heavy	20	19
507.766	816.388	3.5	Tissue-type plasminogen activator.VVPGEEEQK.P00750.y7.light	20	19
561.623	739.885	21.13	Transferrin receptor protein 1.GFVEPDHYVVVGAQR.P02786.y13++.heavy	40	23
558.29	734.88	21.13	Transferrin receptor protein 1.GFVEPDHYVVVGAQR.P02786.y13++.light	40	23
459.59	616.86	25	Transthyretin.GSPAINVAVHVFR.P02766.y11++.heavy	20	18
456.26	611.86	25	Transthyretin.GSPAINVAVHVFR.P02766.y11++.light	20	18
584.327	853.487	10.9	Vascular cell adhesion protein 1.NTVISVNPSTK.P19320.y8.heavy	20	27

580.32	845.47	10.9	Vascular cell adhesion protein 1.NTVISVNPSTK.P19320.y8.light	20	27
489.244	641.313	7.2	Vasorin .ESHVTLASPEETR.PEP20121106020.3.y5.heavy	20	25
485.907	631.304	7.2	Vasorin .ESHVTLASPEETR.PEP20121106020.3.y5.light	20	25
604.819	846.471	23.1	Vitamin K-dependent protein S.SFQTGLFTAAR.PEP20130215010.2.y8.heavy	40	22
599.819	836.471	23.1	Vitamin K-dependent protein S.SFQTGLFTAAR.PEP20130215010.2.y8.light	40	22
716.83	657.335	22.71	Vitronectin.FEDGVLDPDYPR.P04004.y5.heavy	20	38
711.841	647.335	22.71	Vitronectin.FEDGVLDPDYPR.P04004.y5.light	20	38
895.983	1095.59	42	Zinc-alpha-2-glycoprotein.EIPAWVPFDPAAQITK.P25311.y10.heavy	20	40
891.983	1087.59	42	Zinc-alpha-2-glycoprotein.EIPAWVPFDPAAQITK.P25311.y10.light	20	40

Figure S1. The exemplary MRM data (from Skyline) and calibration curves of eleven potential marker serum proteins for BC metastatic progression (alpha-2-macroglobulin, coagulation factor XII, adiponectin, leucine-rich alpha-2-glycoprotein, alpha-2-HS-glycoprotein, Ig mu chain C region, apolipoprotein C-IV, carbonic anhydrase 1, apolipoprotein A-II, apolipoprotein C-II, and alpha-1-acid glycoprotein 1), including sample and LLOQ.

Alpha-2-macroglobulin

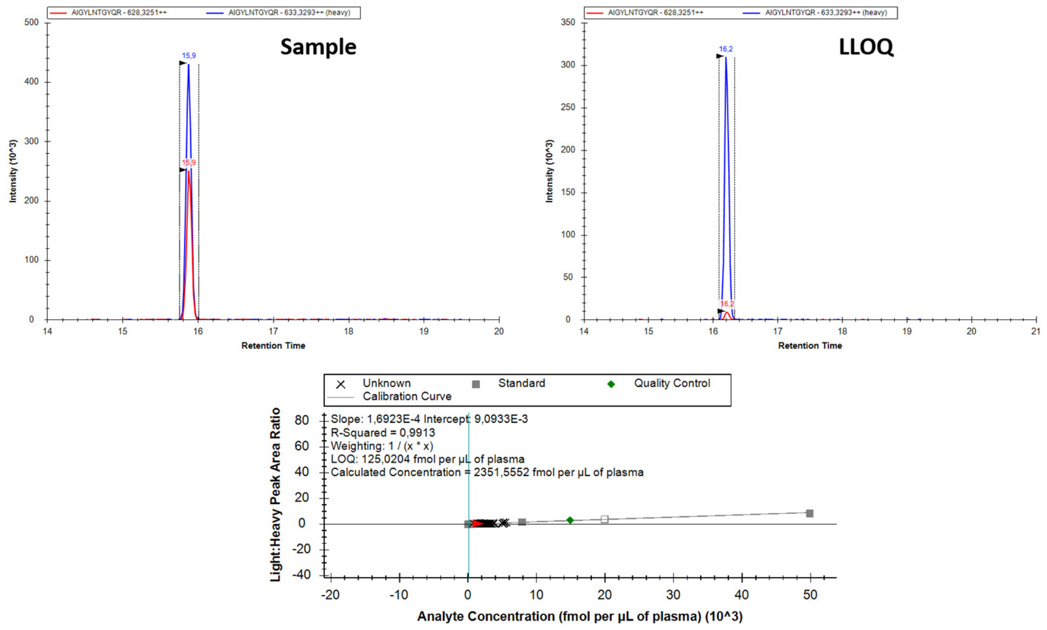

Coagulation factor XII

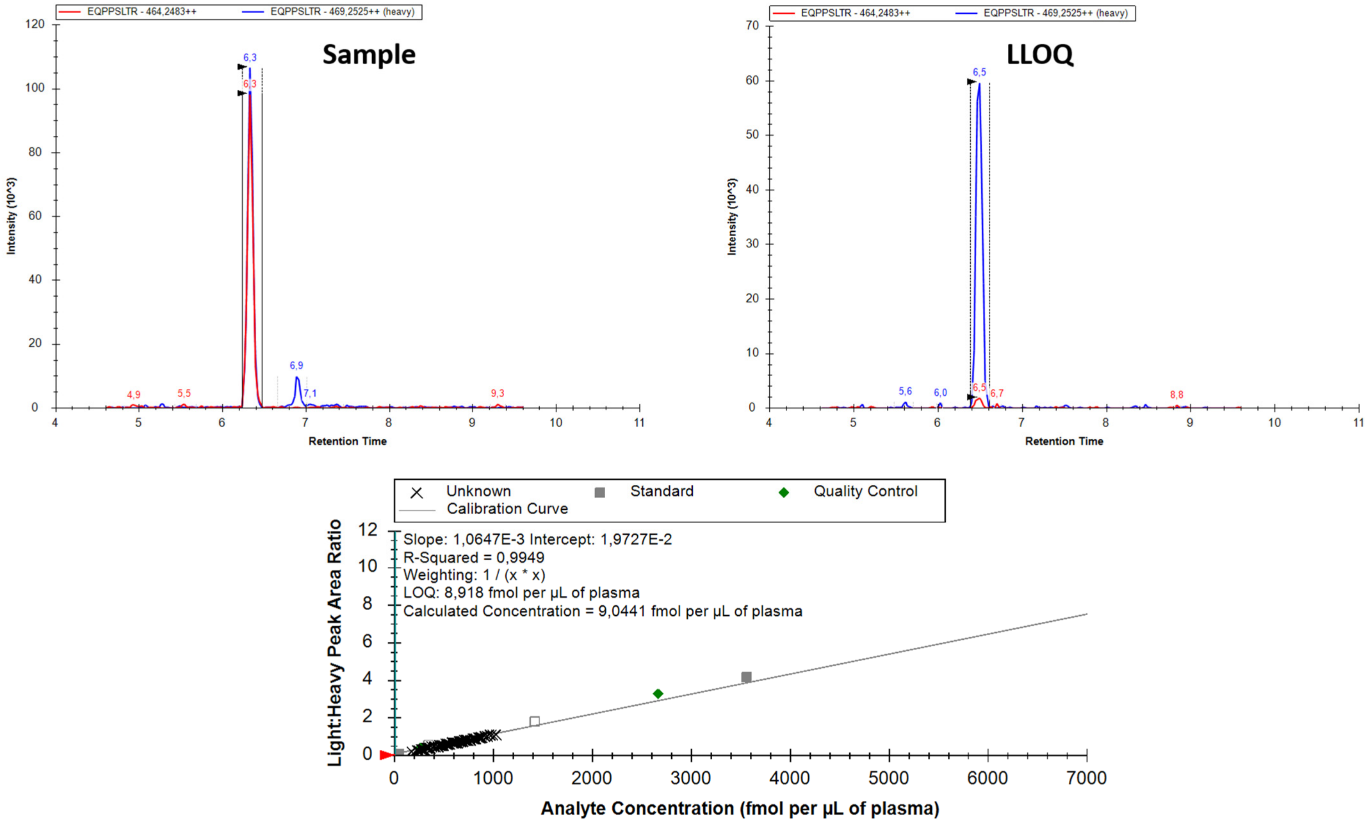

Adiponectin

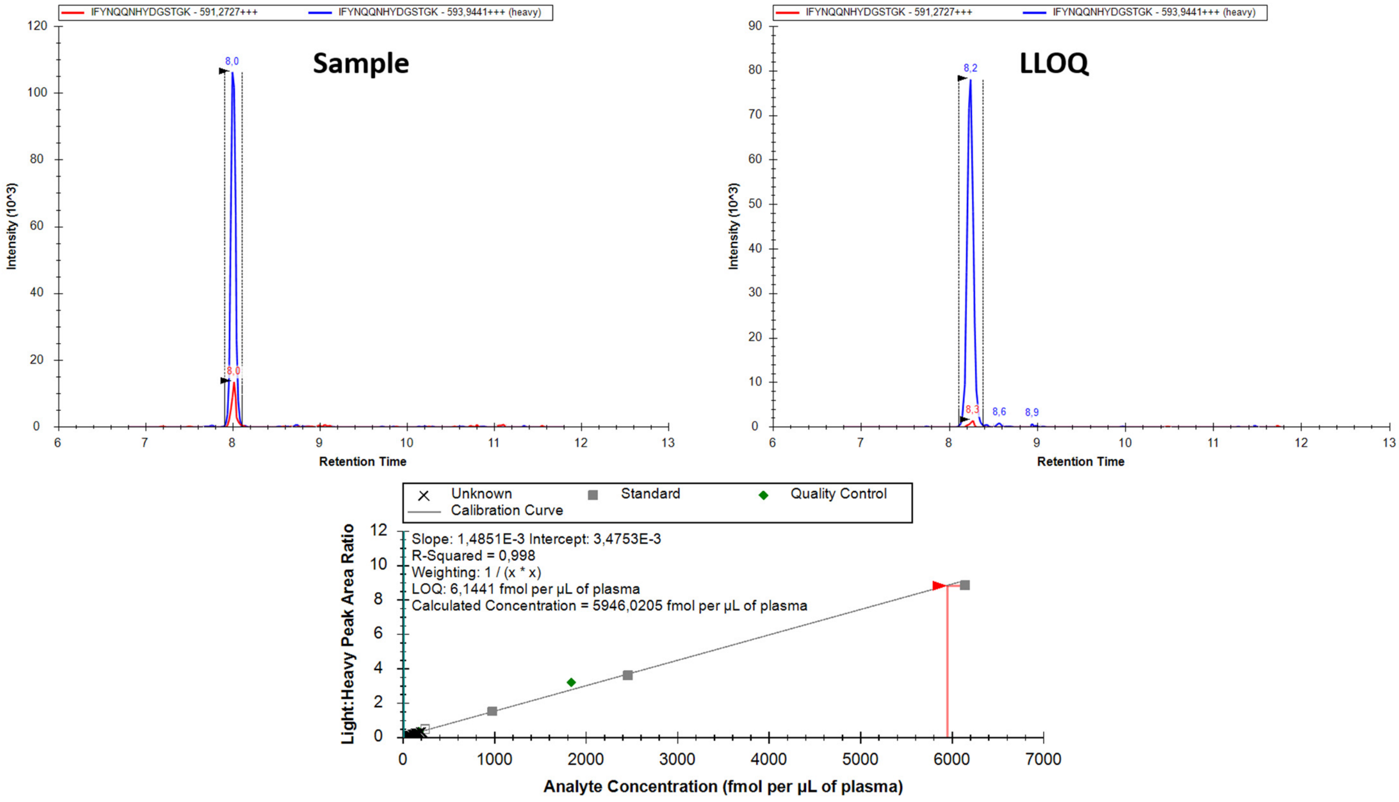

Leucine-rich alpha-2-glycoprotein

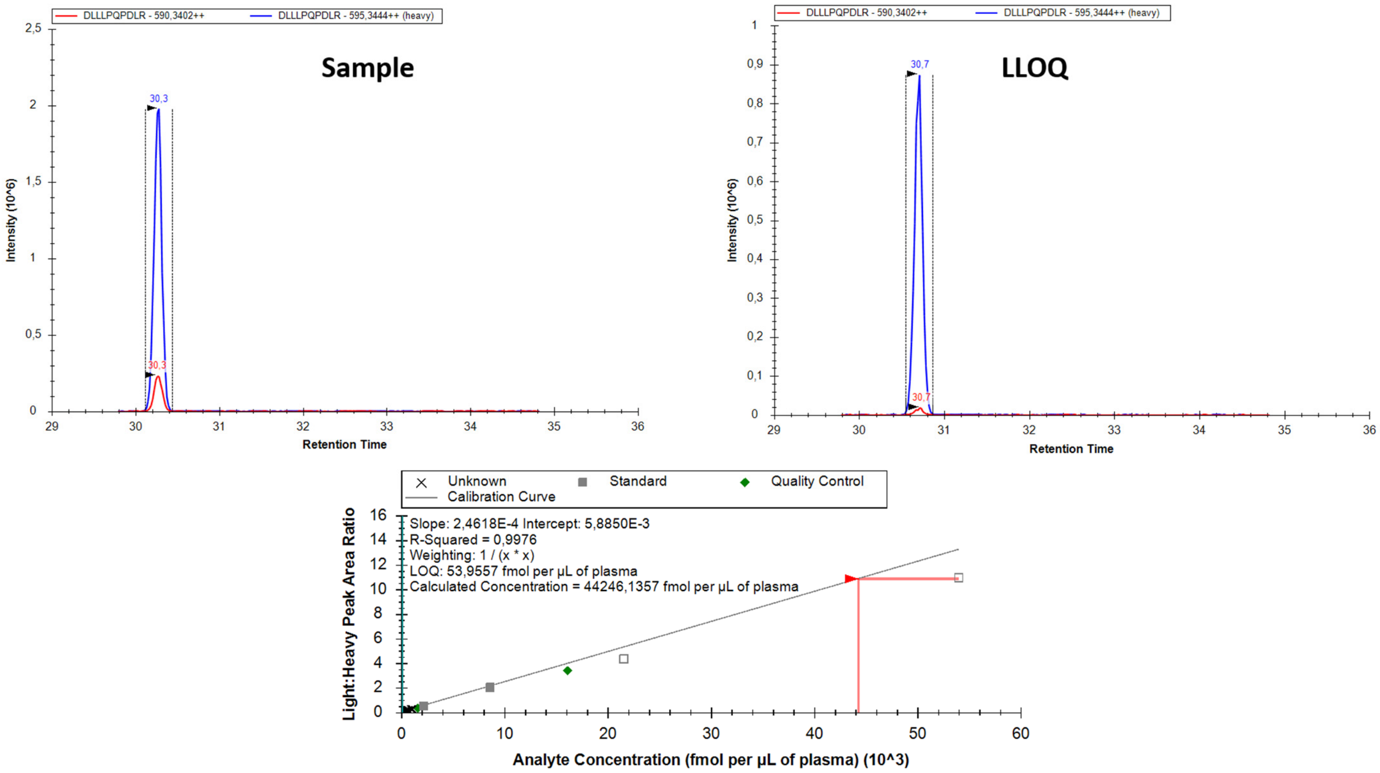

Alpha-2-HS-glycoprotein

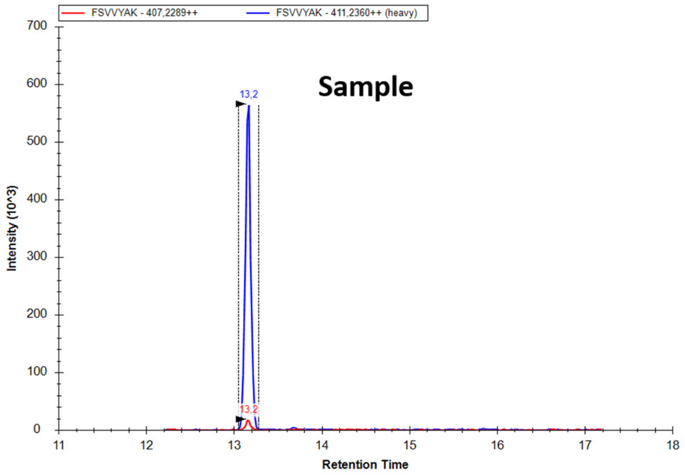

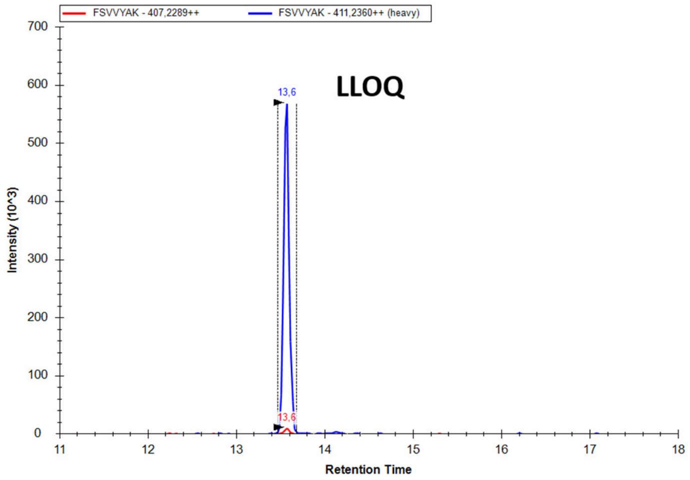

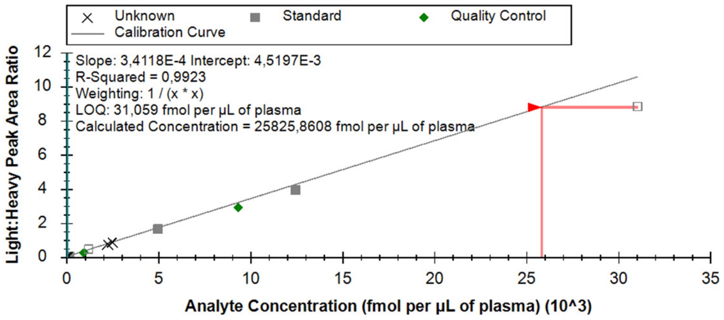

Ig mu chain C region

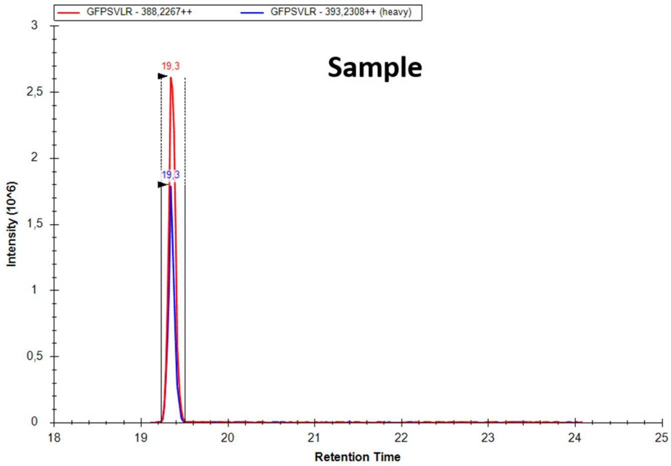

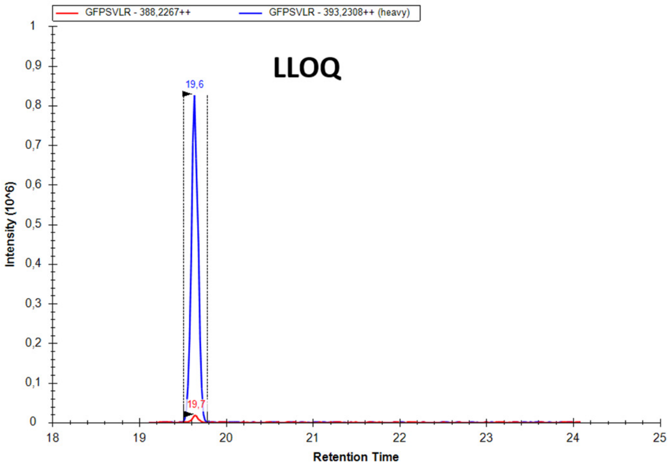

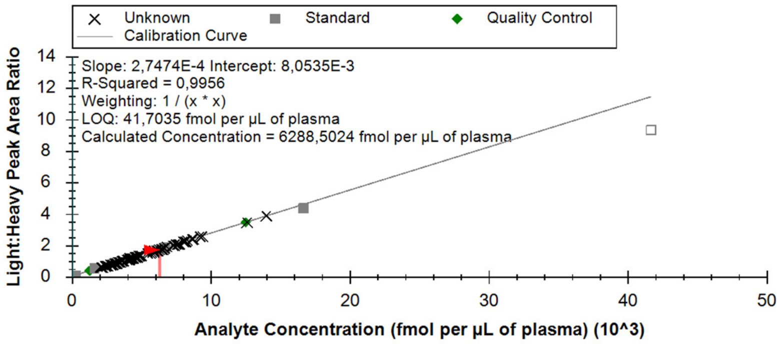

Apolipoprotein C-IV

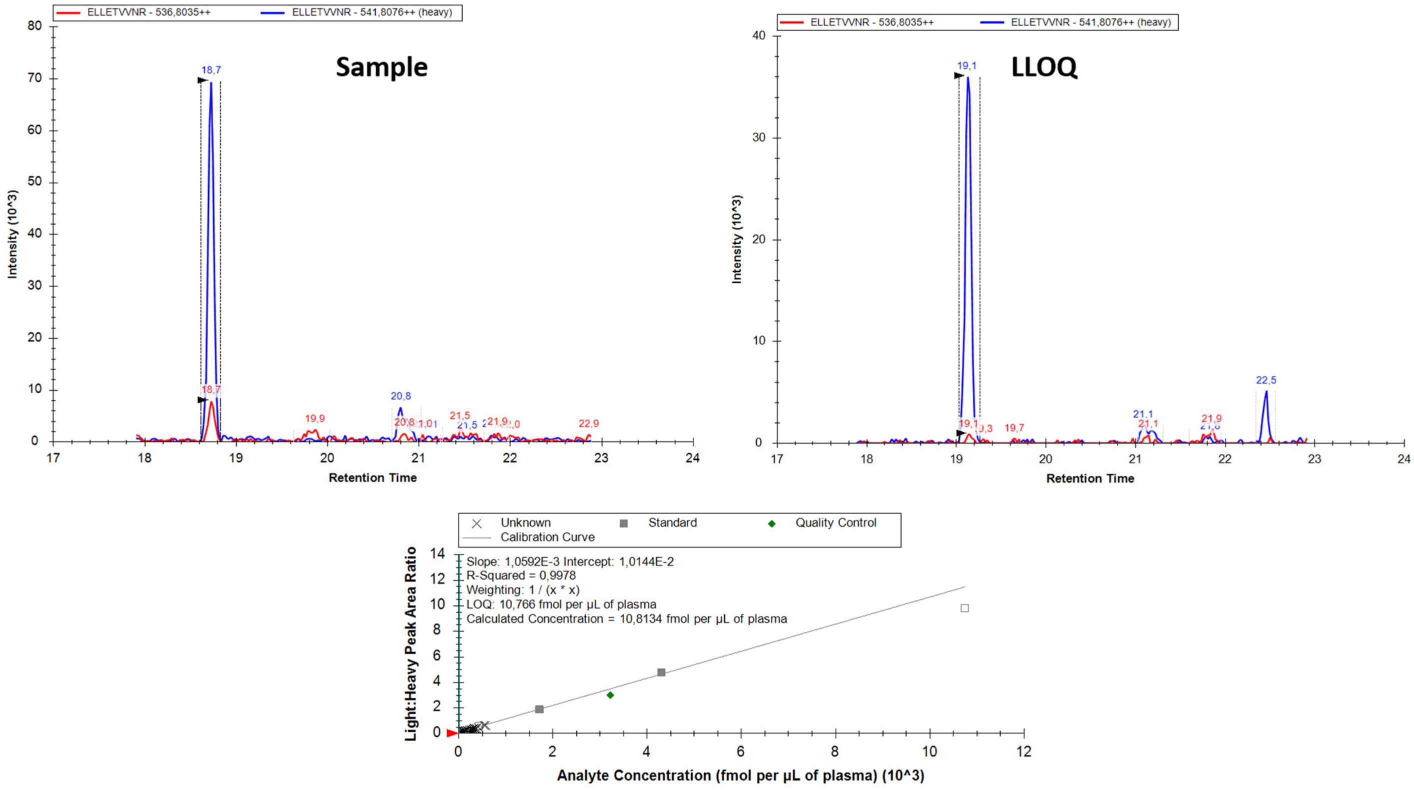

Carbonic anhydrase 1

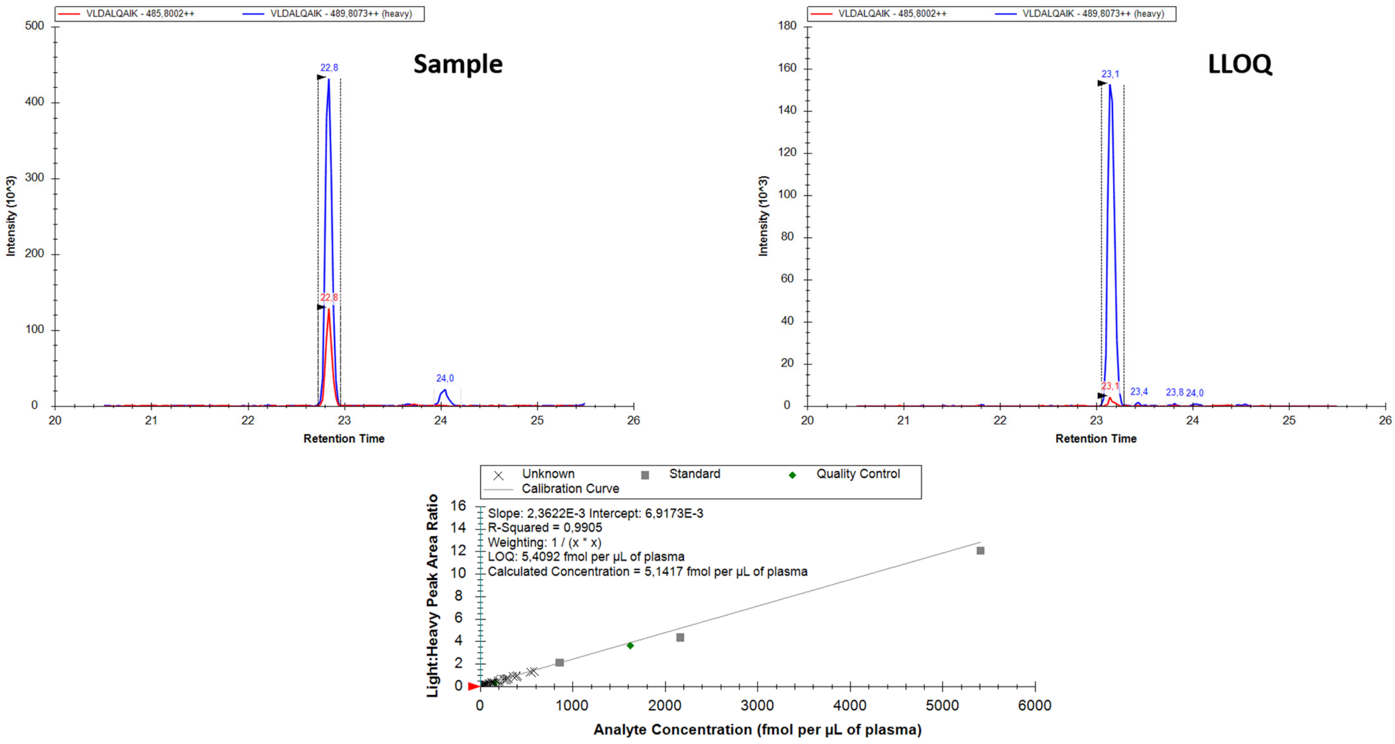

Apolipoprotein A-II

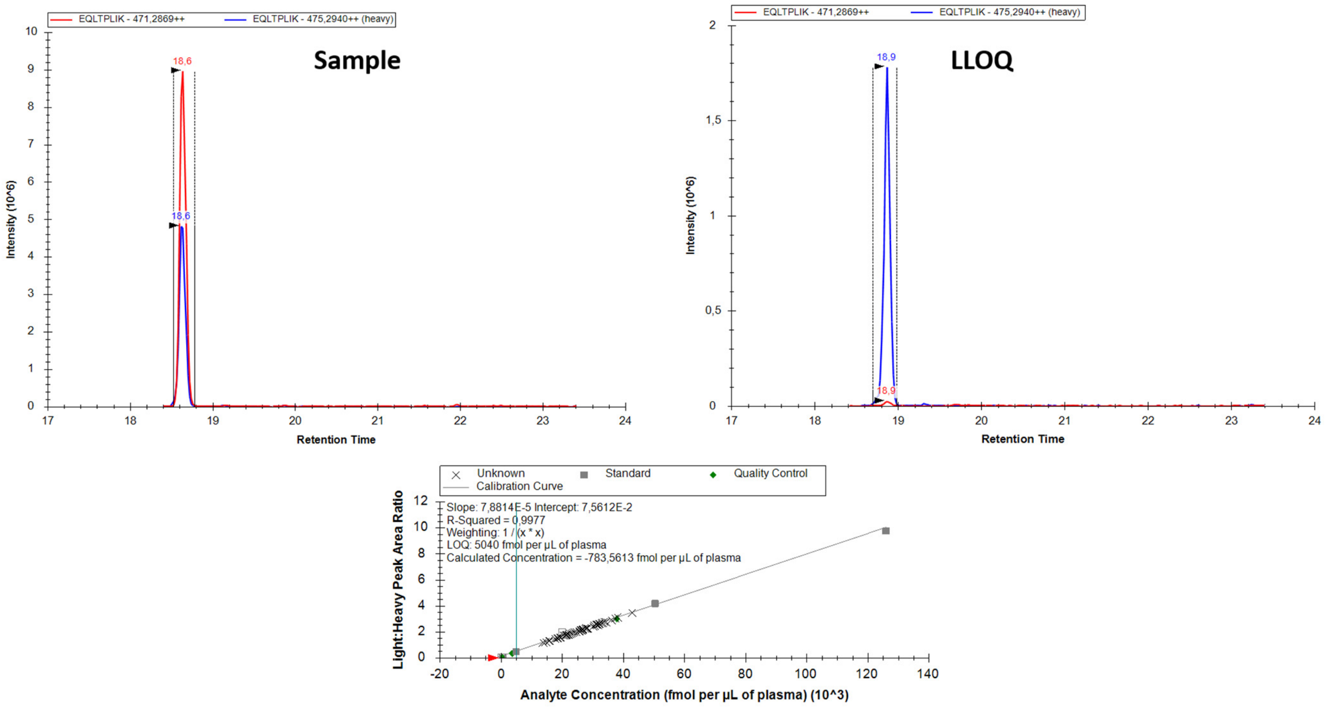

Apolipoprotein C-II

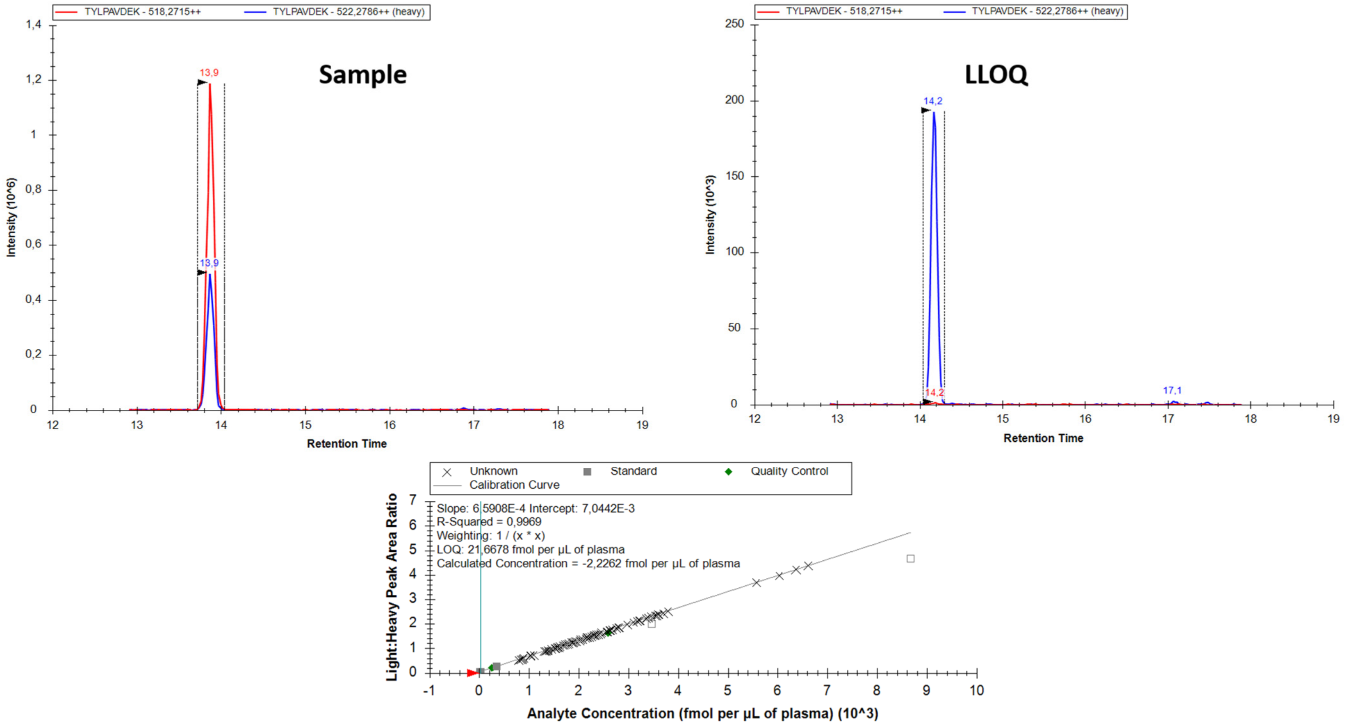

Alpha-1-acid glycoprotein 1

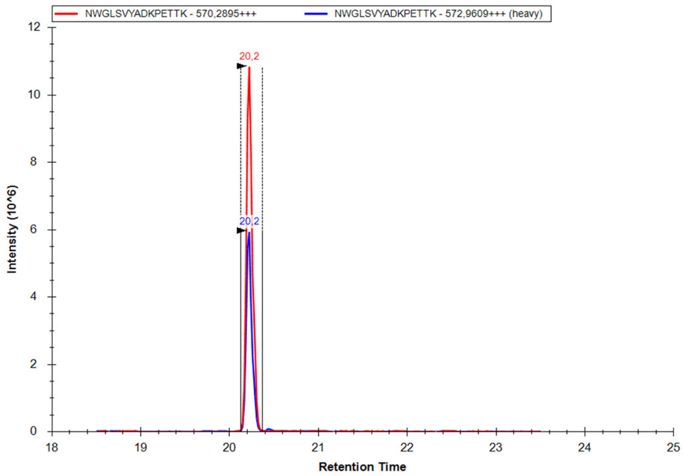

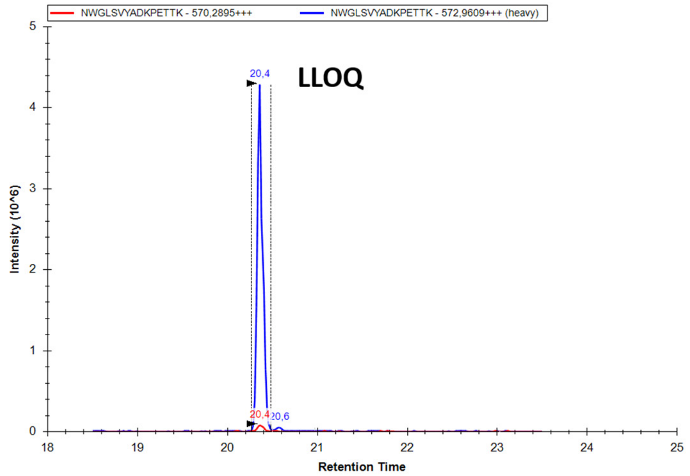

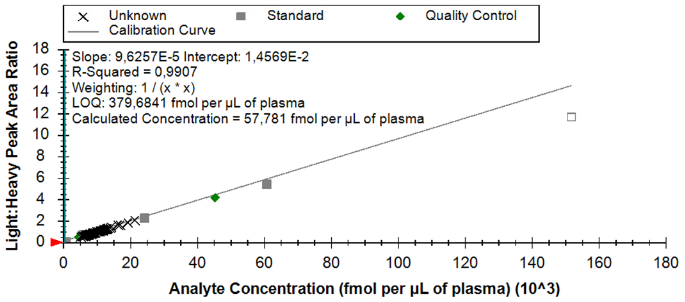

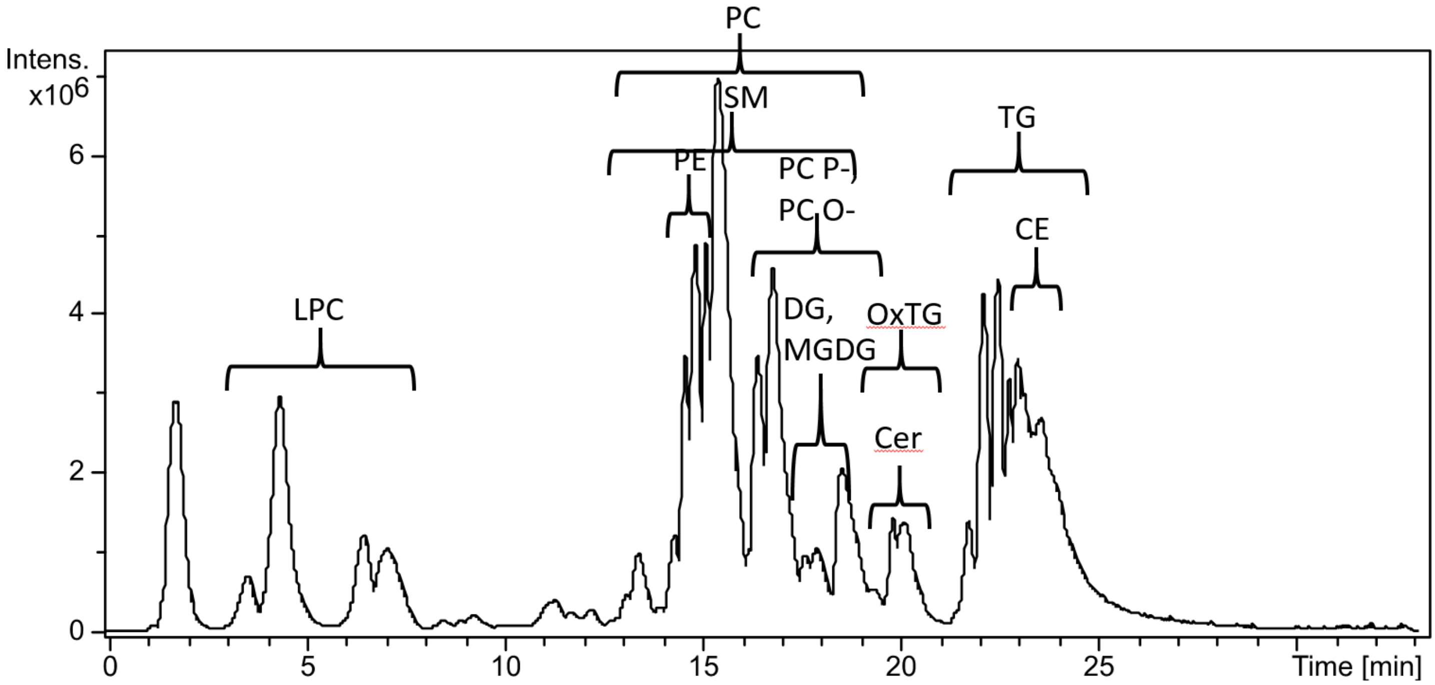

Figure S2. LC-MS chromatogram for lipid extract of the sample with marked regions for each lipid class detected.

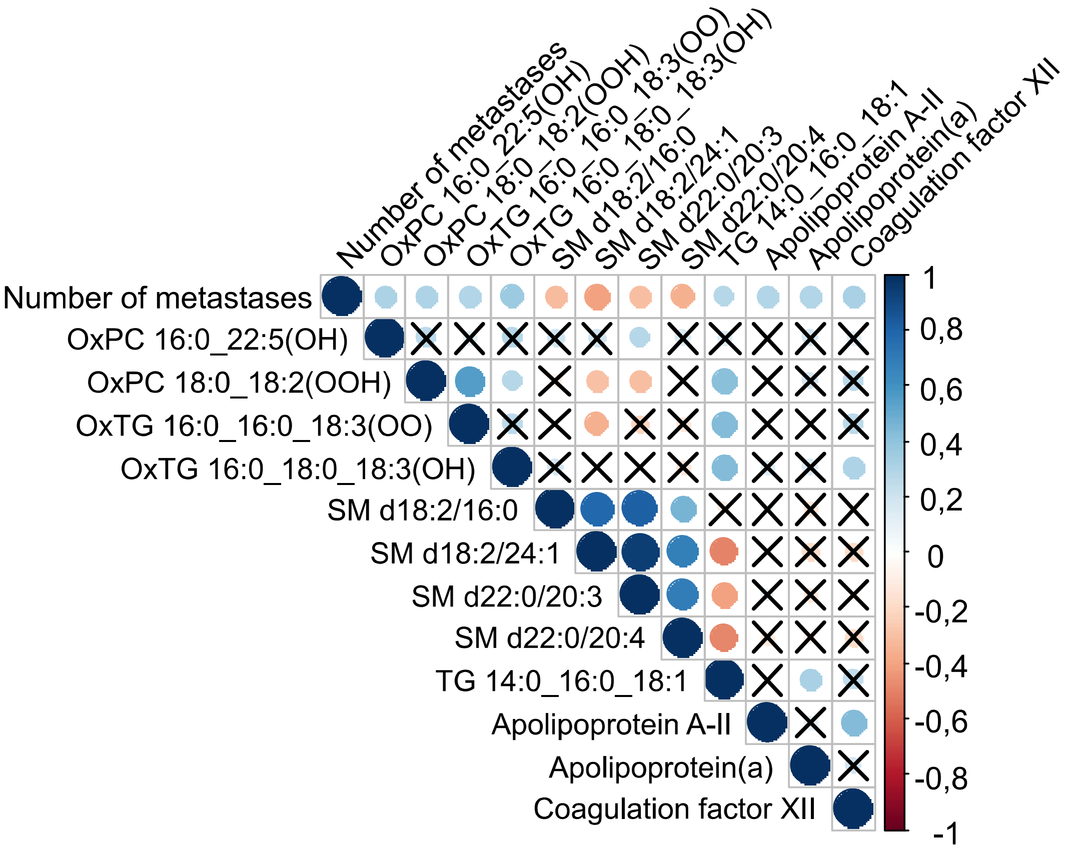

Figure S3. Corrplot of proteins and lipids correlated significantly with number of metastasis.

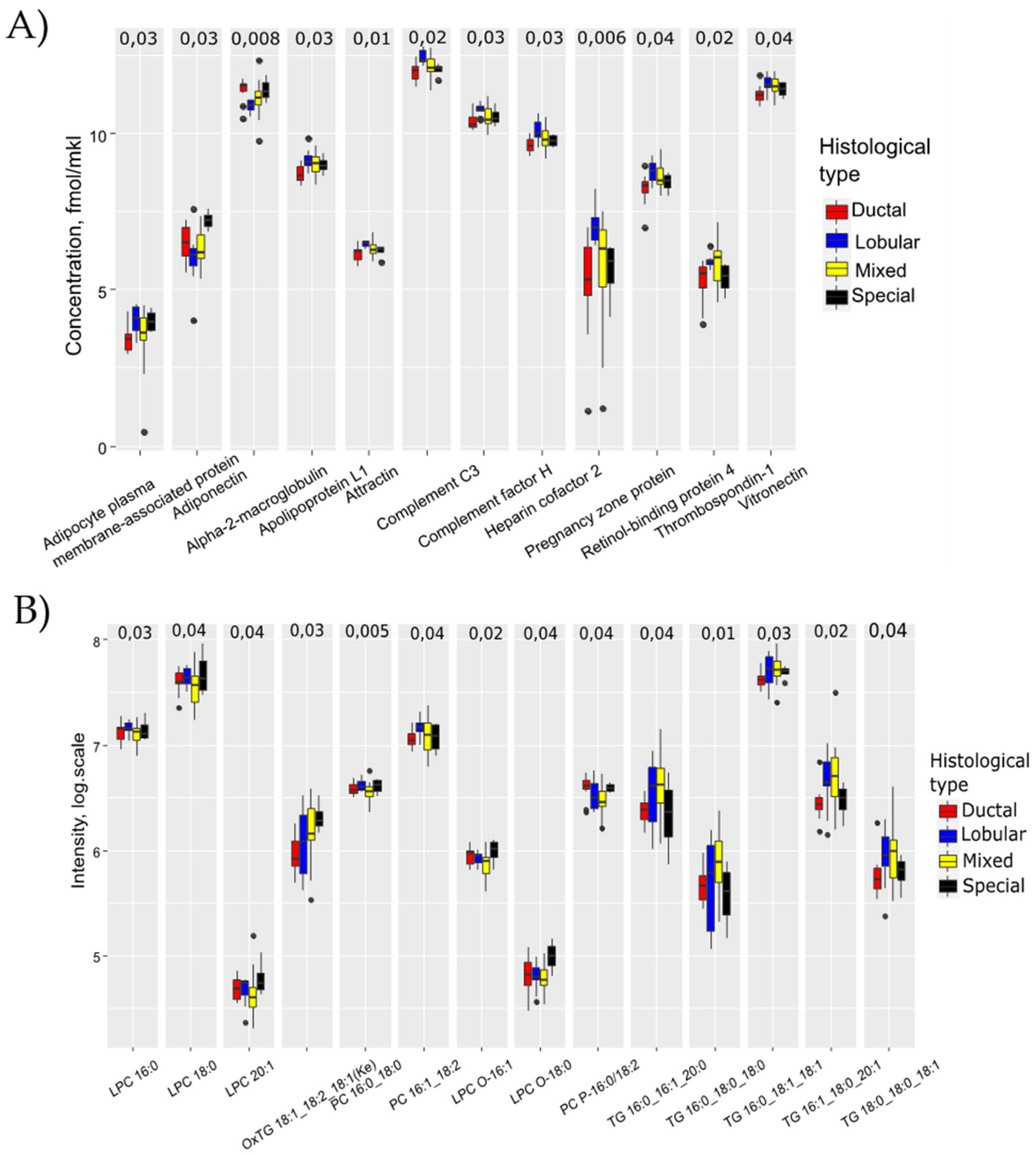

Figure S4. Boxplot of protein and lipids concetrations, which are significantly different in serum in various histological types.

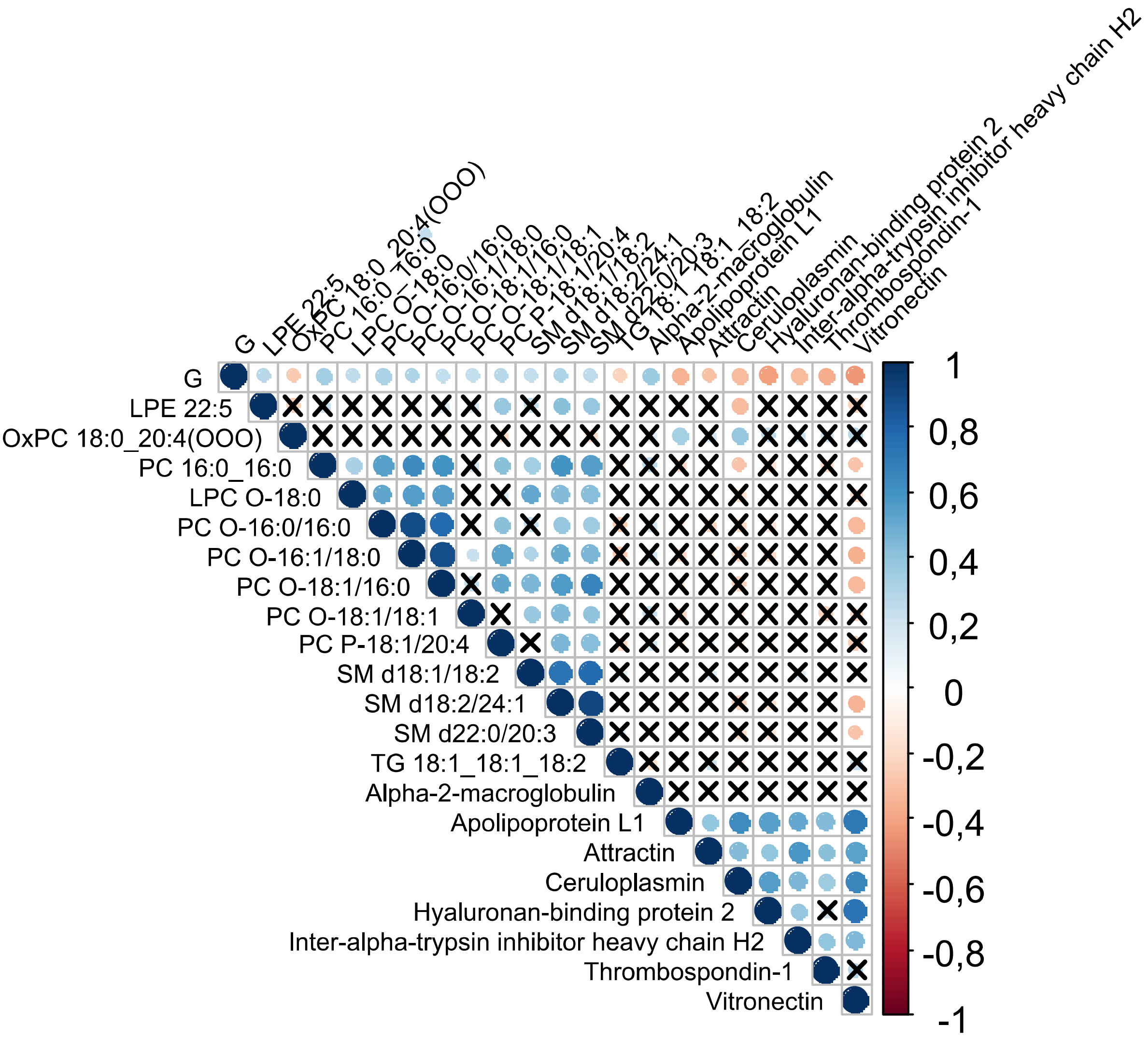

Figure S5. Corrplot of proteins and lipids with significant correlation with BC grade.

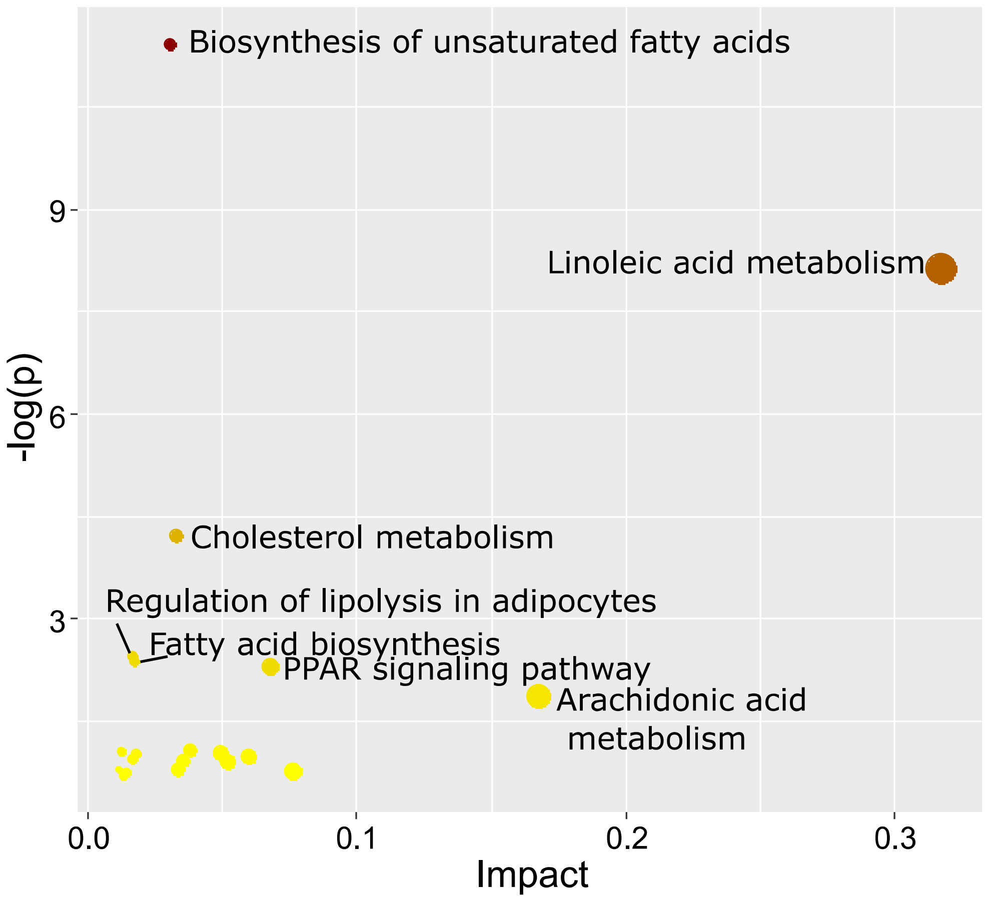

Figure S6. Plot of impact of potential BC metastasis markers on metabolic pathways.

Table S2. Metabolic pathways, disturbed by regional metastasis, including number of all features in the pathway, possibility of zero-impact (p), false discovery rate (FDR), impact on the way.

Pathway	Hits/Total	p	FDR	Impact
Biosynthesis of unsaturated fatty acids	7/79	<0.001	<0.001	0.03
Linoleic acid metabolism	5/57	<0.001	<0.001	0.32
Cholesterol metabolism	3/60	<0.001	0.007	0.03
Regulation of lipolysis in adipocytes	2/69	0.004	0.20	0.02
Fatty acid biosynthesis	2/76	0.004	0.21	0.02
PPAR signaling pathway	2/81	0.005	0.21	0.07
Arachidonic acid metabolism	2/138	0.01	0.51	0.17
Fatty acid elongation	1/67	0.09	1.00	0.01
alpha-Linolenic acid metabolism	1/69	0.09	1.00	0.04
Sphingolipid metabolism	1/72	0.09	1.00	0.05
Fc epsilon RI signaling pathway	1/79	0.10	1.00	0.02
Complement and coagulation cascades	1/80	0.10	1.00	0.06
Fatty acid degradation	1/94	0.12	1.00	0.02
Glycerolipid metabolism	1/99	0.12	1.00	0.05
Fc gamma R-mediated phagocytosis	1/101	0.13	1.00	0.04
Inflammatory mediator regulation of TRP channels	1/135	0.16	1.00	0.03
Platelet activation	1/138	0.17	1.00	0.01
Vascular smooth muscle contraction	1/148	0.18	1.00	0.01
Glycerophospholipid metabolism	1/149	0.18	1.00	0.08
Oxytocin signaling pathway	1/165	0.20	1.00	0.01
