# Supplementary figures and images for: Integrating Proteomics and Lipidomics for Evaluating the Risk of Breast Cancer Progression: A Pilot Study
Source: Biomedicines. 2023 Jun 22;11(7):1786. doi: 10.3390/biomedicines11071786 (PMC10376786; doi:10.3390/biomedicines11071786)

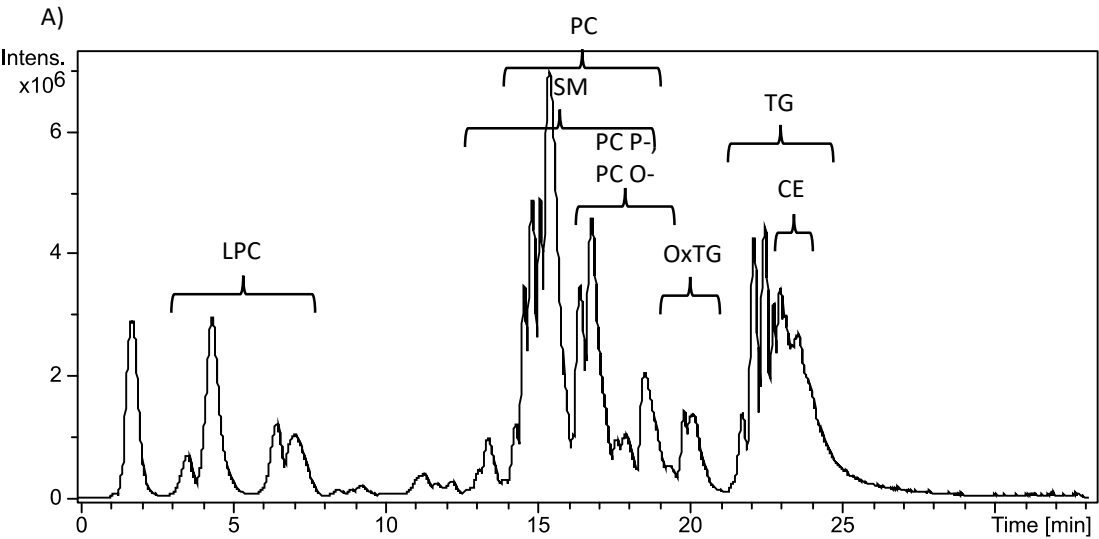

B)

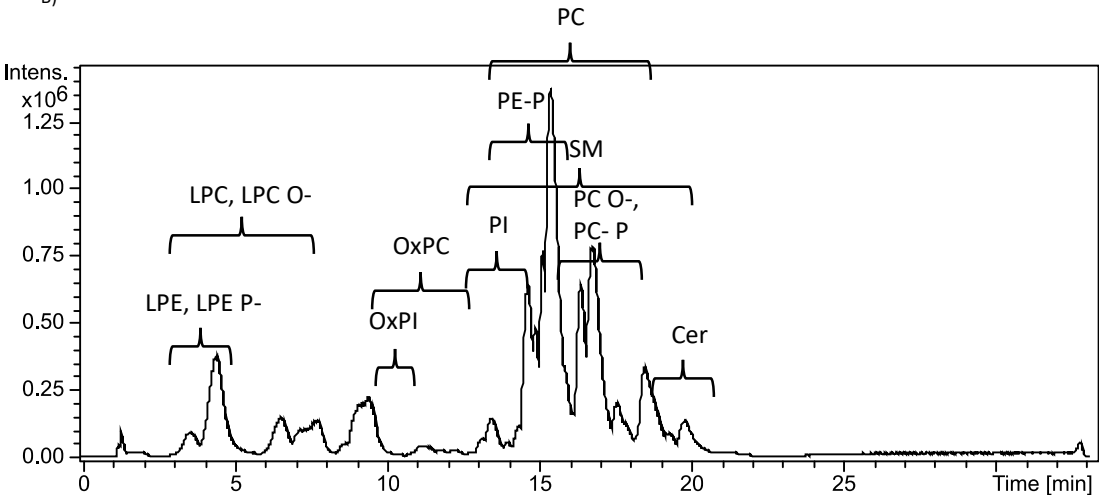

Supplement: Supplementary file 1 [file biomedicines-11-01786-s001.zip › Supplementary S3.pdf]

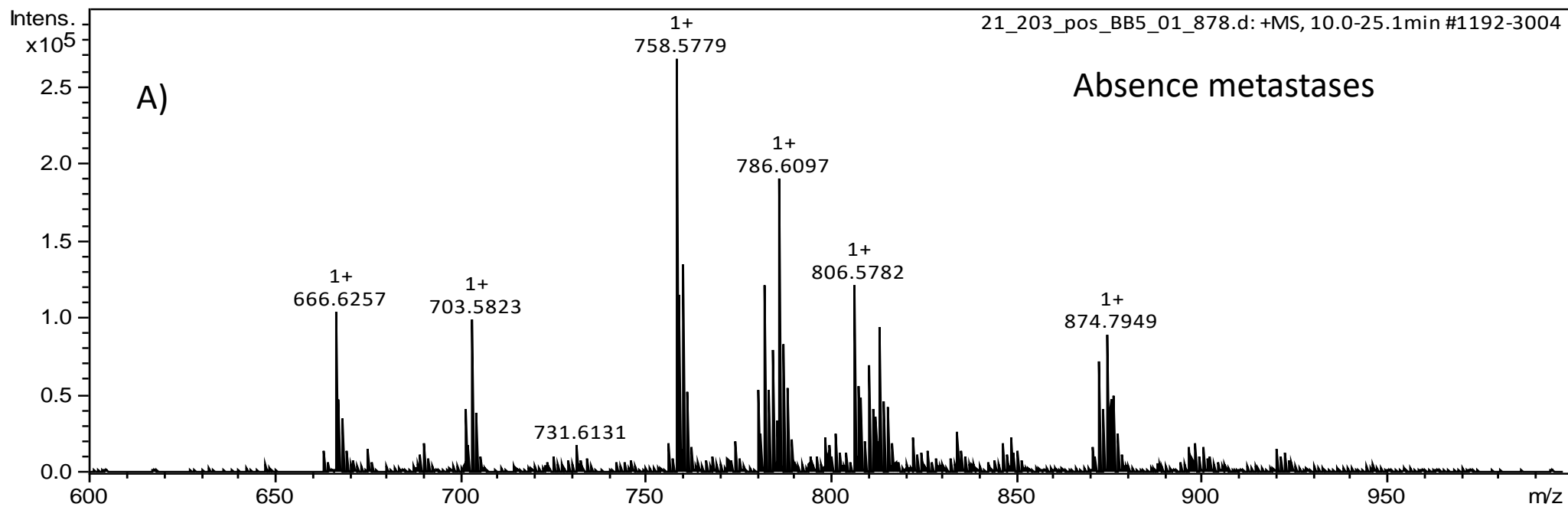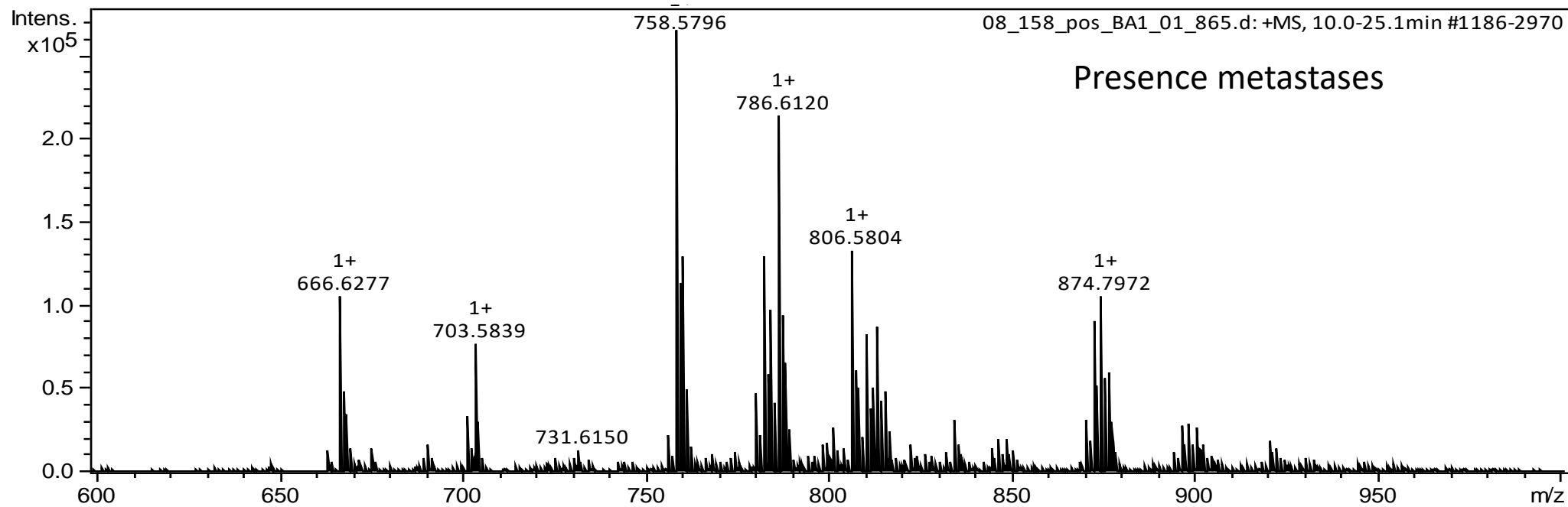

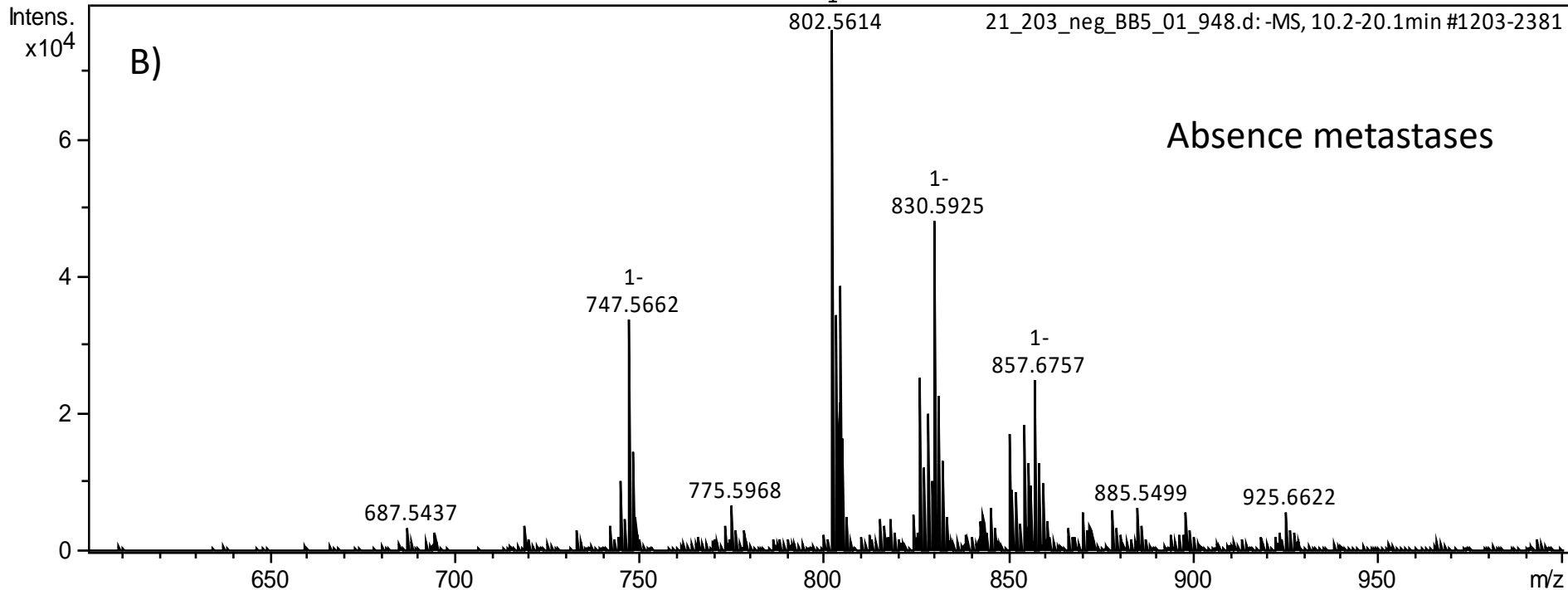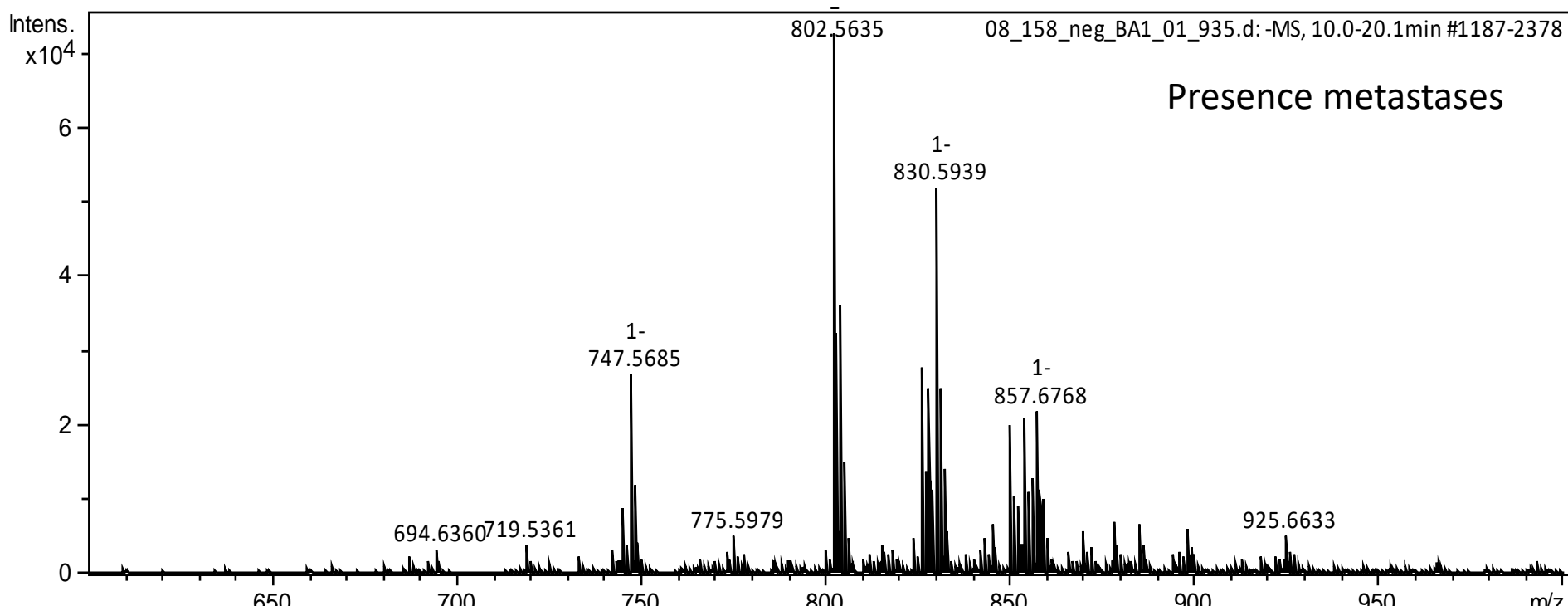

Supplement: Supplementary file 1 [file biomedicines-11-01786-s001.zip › Supplementary S4.pdf]

OxTG 16:0\_18:0\_18:3(OH)

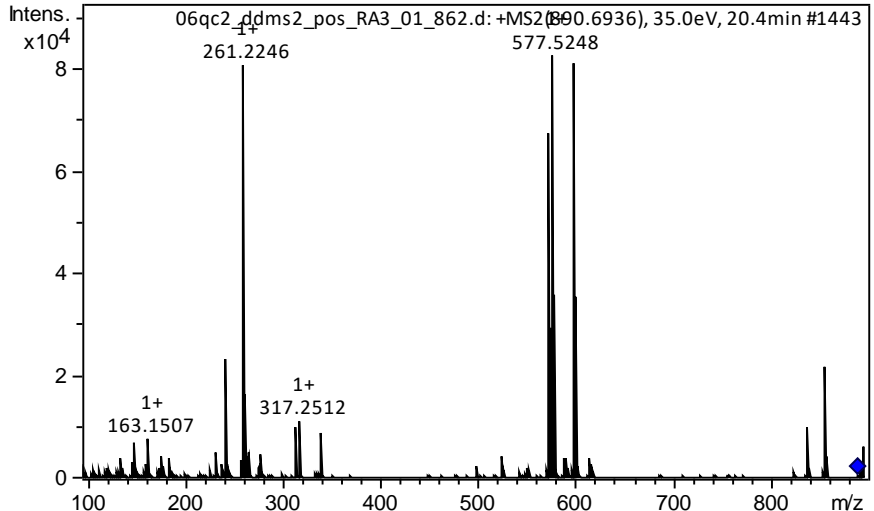

OxTG 18:1\_18:1\_18:1(Ke,OH)

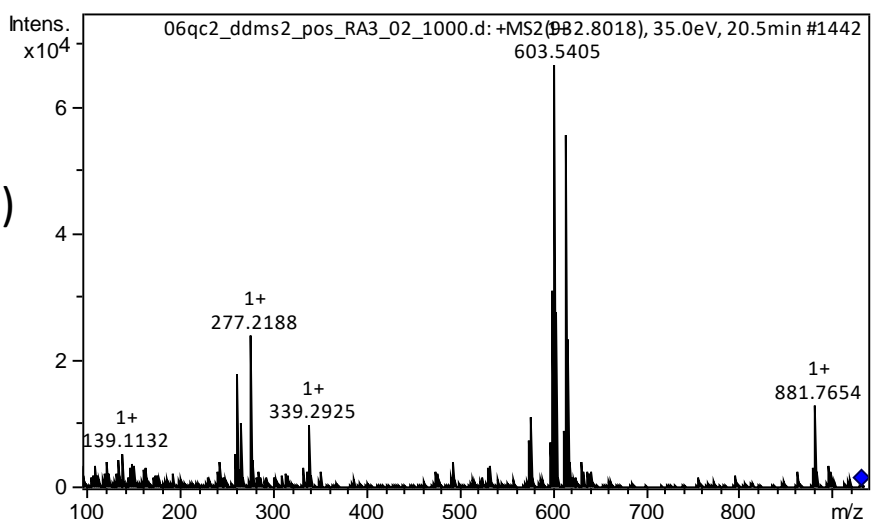

OxTG 18:1\_18:1\_18:2(OOH)

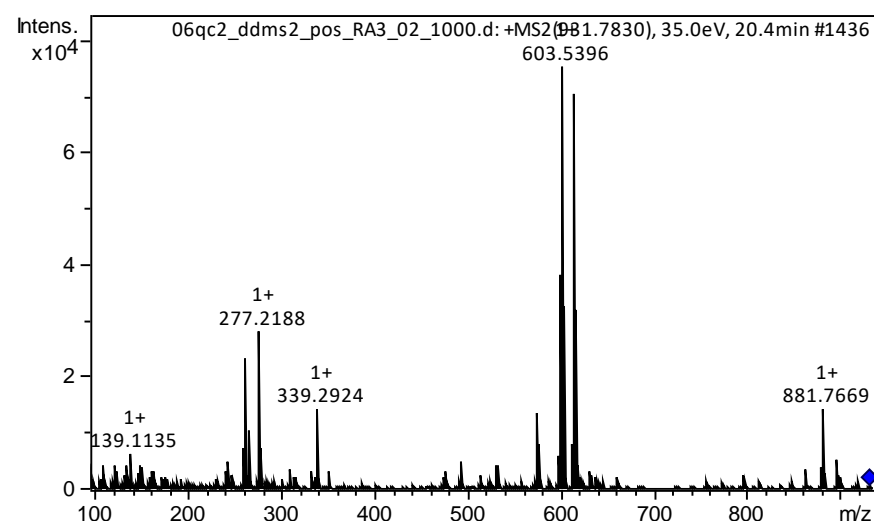

PC 16:1\_20:4

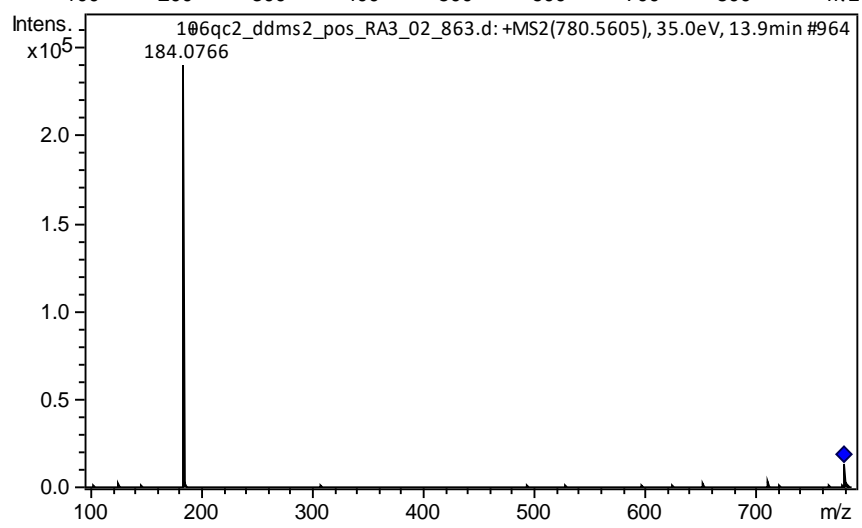

PC 18:0\_22:6

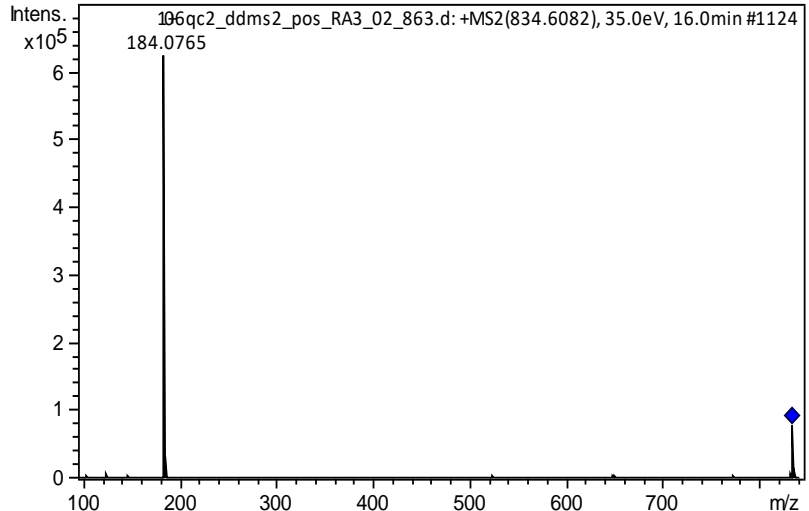

SM d18:2/24:1

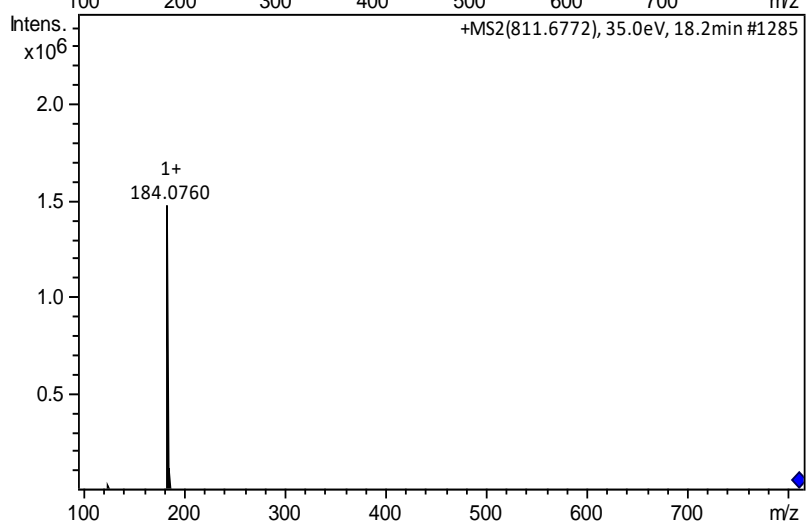

TG 16:0\_16:1\_18:1

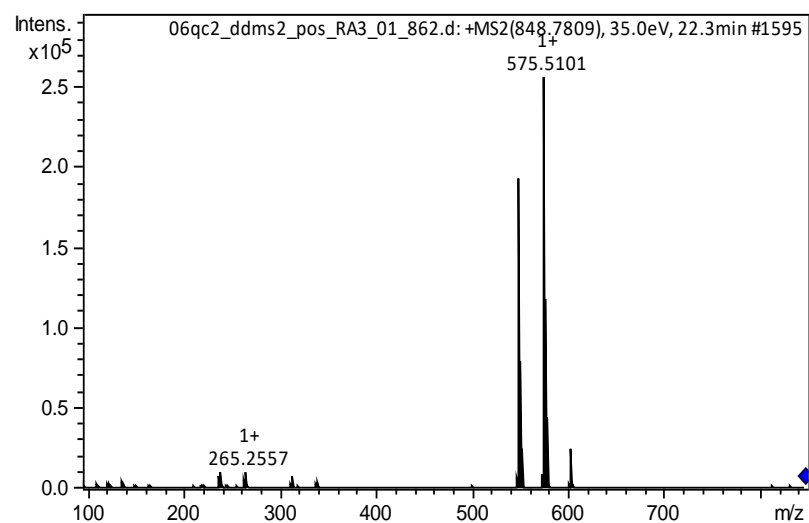

TG 18:1\_18:1\_18:2

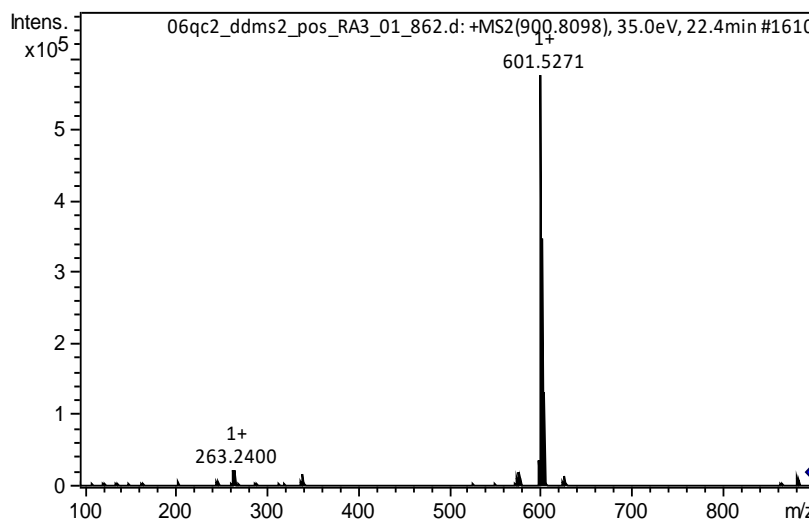

Supplement: Supplementary file 1 [file biomedicines-11-01786-s001.zip › Supplementary S5.pdf]
